# ^15^N labeling and analysis of ^13^C–^15^N and ^1^H–^15^N couplings in studies of the structures and chemical transformations of nitrogen heterocycles

**DOI:** 10.1039/c9ra04825a

**Published:** 2019-08-28

**Authors:** Sergey L. Deev, Igor A. Khalymbadzha, Tatyana S. Shestakova, Valery N. Charushin, Oleg N. Chupakhin

**Affiliations:** Ural Federal University 19 Mira Street 620002 Yekaterinburg Russian Federation deevsl@yandex.ru; I. Ya. Postovsky Institute of Organic Synthesis 22 S. Kovalevskoy Street 620219 Yekaterinburg Russian Federation

## Abstract

This review provides a generalization of effective examples of ^15^N labeling followed by an analysis of ^13^C–^15^N (*J*_CN_) and ^1^H–^15^N (*J*_HN_) coupling constants in solution as a tool to study the structural aspects and pathways of chemical transformations (*e.g.*, rearrangements and ring-chain tautomerisms) in monocyclic and fused nitrogen heterocycles. This approach allows us to significantly expand and supplement the scope of NMR techniques for heterocyclic compounds. Moreover, methods for the incorporation of ^15^N atoms into the cores of various N-heterocycles have been collected in this work.

## Abstract 2

This review provides a generalization of effective examples of ^15^N labeling followed by an analysis of *J*_CN_ and *J*_HN_ couplings in solution as a tool to study the structural aspects and pathways of chemical transformations in nitrogen heterocycles.

## Graphical abstract

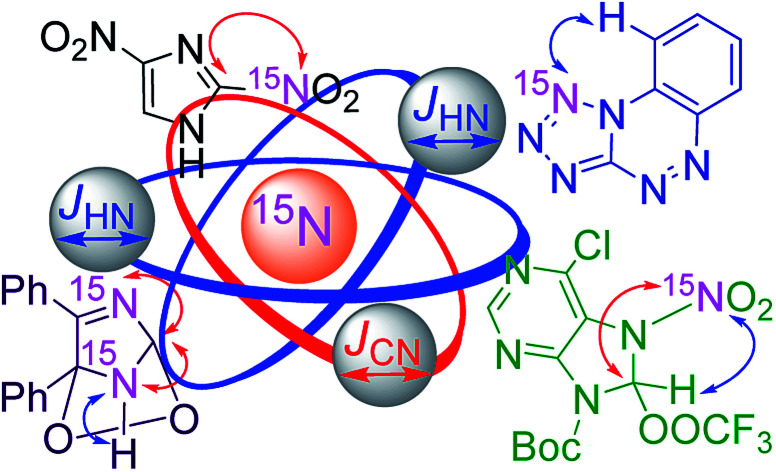

## 1. Introduction

Nitrogen heterocycles are a ubiquitous class of organic compounds that include azirine, azetidine, azole, azine and azepine derivatives and their fused analogs. These structures are inherent in drug design^1–7^ and natural compounds,^8,9^ in catalysis for cross-coupling and asymmetric synthesis reactions,^10,11^ in materials science as metal complexes with luminescent properties,^12–14^ in ligands for the separation of lanthanides^15^ and in high-density energy materials.^16,17^

At first glance, it seems that establishing the structure of poly-nitrogen-containing compounds can be achieved by general NMR methods suitable for carbon-containing compounds; however, this statement is not correct. It should be noted that heterocycles have low densities of hydrogen and carbon atoms. Therefore, the application of conventional NMR methods (1D ^1^H and ^13^C spectroscopy, 2D HMQC, HMBC, INADEQUATE, *etc.*) might be ineffective for the structural estimation of poly-nitrogen compounds. Another important consequence of an increased nitrogen atom content is a decrease in the aromaticity and the tendency to undergo ring-chain transformations. Thus, the confirmation of the structure can be complicated by the azide–tetrazole equilibrium, ANRORC (Addition of Nucleophile, Ring Opening, Ring Closure) reaction, Dimroth rearrangement, *etc.*, which are observed in the azolo and azine series under the action of nucleophilic reagents and solvents. In this case, researchers usually use X-ray crystallography or comparison of UV-vis and ^1^H and ^13^C NMR spectra with the data of model compounds with unambiguously confirmed structures. Unfortunately, the first approach gives information about the structure of compounds only in the solid state and is not suitable for the analysis of mixtures of compounds. The second method allows for the determination of structural characteristics in solution, but it can lead to incorrect conclusions.^18^ There are several approaches to solve these issues. For example, the use of 2D H–(C)–N multiple bond correlation (HCNMBC) experiments was described for the structural confirmation of *N*-alkylated azolo derivatives based on the natural ^15^N abundance.^19,20^ However, these NMR procedures rely on magnetization transfer through ^13^C–^15^N *J*-coupling, and although they allow the determination of some other structures of simple monocyclic azoles, they cannot be considered as general.

The selective ^15^N-labeling of organic molecules leads to the appearance of additional ^1^H–^15^N and ^13^C–^15^N spin–spin coupling constants (SSCCs) that significantly expand the application of NMR methods in the determination of molecular structures. This approach is widely used in the chemistry of proteins and nucleic acids.^21–24^ Although ^15^N-labeling and the subsequent analysis of *J*_HN_ and *J*_CN_ couplings in structural studies of nitrogen heterocycles have been described in a few articles, such a method can be effective for the determination of the structure and studying of the mechanism of chemical transformations of azoles, azines and their fused derivatives. Most of these articles were presented in early published review papers and books that described the highlight achievements in various areas of chemistry.

However, the incorporation of a ^15^N atom into structures leads to the appearance of isotope shifts.^25^ These data may only be considered as additional features to confirm the structure. The chemical shifts of labeled nitrogens in 1D ^15^N NMR spectra can also be used as additional characteristics of enriched compounds.^26^ However, information on chemical shifts is relative and can only be used in the context of the already obtained detailed data. In contrast, the measurement of ^13^C–^15^N and ^1^H–^15^N coupling constants provides unambiguous information about the molecular structures.

Despite the great opportunities for selective ^15^N incorporation followed by an analysis of *J*_HN_ and *J*_CN_ couplings, no systematic review devoted to this tool for structural studies of heterocycles has been presented until today. This is the first attempt to generalize the literature data on ^15^N-labeling and show the capacity of the usage of *J*_HN_ and *J*_CN_ couplings for the determination of the molecular structure and the chemical transformations of nitrogen-containing heterocycles. The relevance of the topic has been confirmed by a recently published article describing the Dimroth-type ring transformation of an azine fragment in the imidazo[1,2-*a*]pyrimidine scaffold, which is of significant interest for modern medicinal chemistry.^27^ The authors showed the efficiency and universality of the use of ^15^N-labeling and the analysis of ^1^H–^15^N coupling constants for ring-chain transformations, which require more detail to determine the structures of heterocyclic compounds. It was this work that drove us to summarize the literature data on the incorporation of ^15^N isotopes and the use of *J*_HN_ and *J*_CN_ couplings in the chemistry of heterocycles. Analysis of the literature showed that this approach is general and covers different series of heterocyclic systems.

This review includes three sections. The first section describes the application of ^1^H–^15^N and ^13^C–^15^N constants for confirmation of the ways to fuse azole, azine and azepine rings during the synthesis of bicyclic, tricyclic and polycyclic compounds and the use of *J*_HN_ and *J*_CN_ in the studies of tautomeric rearrangements of heterocyclic derivatives. The next section reports the ^15^N-labeling and analysis of ^1^H–^15^N and ^13^C–^15^N spin–spin interactions in the determination of the mechanism of ring transformation rearrangements. The last section includes information about the use of *J*_HN_ and *J*_CN_ couplings for the determination of the sites and mechanisms of interaction of heterocycles with electrophilic and nucleophilic reagents, which occurs without the transformation of the heterocyclic framework.

## 2. Use of *J*_HN_ and *J*_CN_ for establishing methods for the heterocyclization of azole, azine or azepine to azole and azine fragments and studying ring-chain tautomerism in a series of heterocycles

A combination of ^15^N-labeling and an analysis of *J*_HN_ and *J*_CN_ couplings is one of the approaches for the confirmation of the structure of a fused heterocyclic system that can be formed by different methods for the cyclization of an azole, azine or azepine ring to various five- or six-membered heterocycles. Moreover, this method may be effective for the study of ring-chain tautomerisms that are observed in various classes of heterocycles.

### 2.1. Determination of methods of heterocyclization based on an analysis of ^1^H–^15^N and ^13^C–^15^N spin–spin interactions

**Scheme 1:**
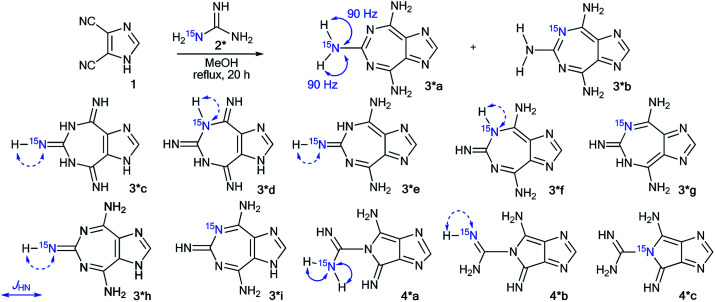
Establishing the structure of the condensation product of 3,4-dicyanoimidazole with guanidine. The observed ^1^*J*_HN_ couplings in the ^1^H NMR spectra are shown by blue arrows. The expected couplings (^1^*J*_HN_) in the excluded structures are indicated by dashed arrows.

The measurement of ^13^C–^15^N and ^1^H–^15^N spin–spin interactions can be used for the determination of alternative heterocyclization pathways, including monocyclic derivatives. This method can be efficient even for the analysis of isotopomer mixtures. For example, the interaction of 4,5-dicyanoimidazole 1 with ^15^N-guanidine 2* led to compounds 3*a and 3*b (Scheme 1).^28^ It should be noted that the reaction could give tautomers 3*a–i and isomers 4*a–c. Nonetheless, the registration of the signals of the two labeled nitrogen nuclei as a triplet and singlet in the ^15^N NMR spectrum provided solid evidence of the formation of isotopomers 3*a and 3*b and excluded the formation of tautomers 3*c–i. Furthermore, structure 3*a was confirmed by observation of the single ^1^H–^15^N spin–spin coupling (^1^*J*_HN_ = 90 Hz) between a ^15^N atom and the protons of an NH_2_ group in the ^1^H NMR spectrum. If a mixture of compounds 4*a–c were produced, additional ^1^*J*_HN_ splitting for the ^15^NH imino group would appear in both the ^1^H and ^15^N NMR spectra. However, such ^1^H–^15^N SSCCs were absent, which excluded the formation of 4*a–c. This result also showed that the measurement of ^1^*J*_HN_ couplings can be effective in the determination of the structure of prototropic tautomers instead of an analysis of the chemical shifts of ^13^C and ^15^N atoms in the NMR spectra.^29–31^

**Scheme 2:**
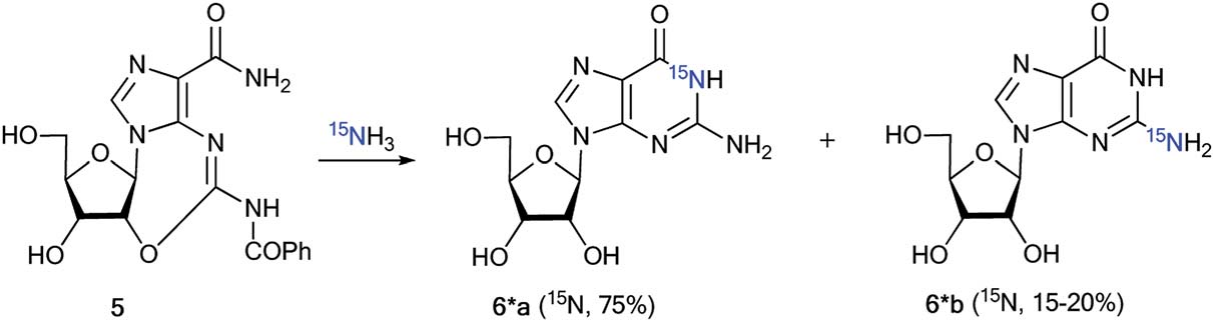
Synthesis of ^15^N-labeled guanosines 6*a and 6*b using ^15^NH_3_.

**Scheme 3:**
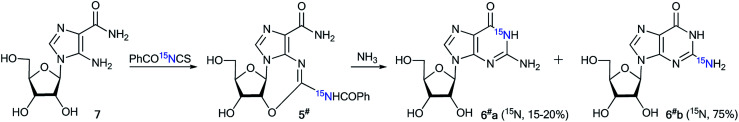
Synthesis of ^15^N-labeled guanosines 6*a and 6*b using ^15^N-benzoyl isothiocyanate.

An analysis of direct ^13^C–^15^N coupling constants allowed for the determination of the method of cyclization in the reaction of guanosine with glycidaldehyde.^32^ Two methods were used for the incorporation of ^15^N atoms in the pyrimidine fragment of guanosine. The first procedure was based on the treatment of compound 5 with [^15^N]-ammonia (Scheme 2). This approach gave an isotopomeric mixture containing 75% 6*a and 15–20% 6*b. Another synthesis of 6^#^a and 6^#^b that included obtaining the ^15^N-labeled amide 5^#^ by the interaction of [^15^N]-benzoyl isothiocyanate and imidazolecarboxamide derivative 7 is depicted in Scheme 3. The reaction of compound 5^#^ with ammonia yielded a mixture containing 6^#^a (15–20%) and 6^#^b (75%).

**Scheme 4:**
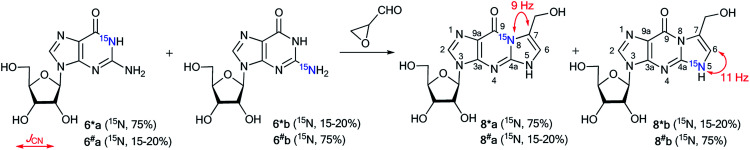
Reaction of guanosines 6*a and 6*b with glycidaldehyde. The observed ^1^*J*_CN_ couplings in the ^13^C NMR spectra are shown by red arrows.

The use of the mixture of ^15^N-guanosines 6*a/6^#^a and 6*b/6^#^b in a reaction with glycidaldehyde led to obtaining samples that had different concentrations of isotopomers 8*a (^15^N, 75%)/8^#^a (^15^N, 15–20%) and 8*b (^15^N, 15–20%)/8^#^b (^15^N, 75%) (Scheme 4). Differences in the enrichment of products 8*a/8^#^a and 8*b/8^#^b allowed for the direct measurement of ^13^C–^15^N coupling constants (^1^*J*_C7–N8_ 9 Hz and ^1^*J*_C6–N5_ 11 Hz) and the assignment of the C6 and C7 signals in the ^13^C NMR spectra.

**Fig. 1:**
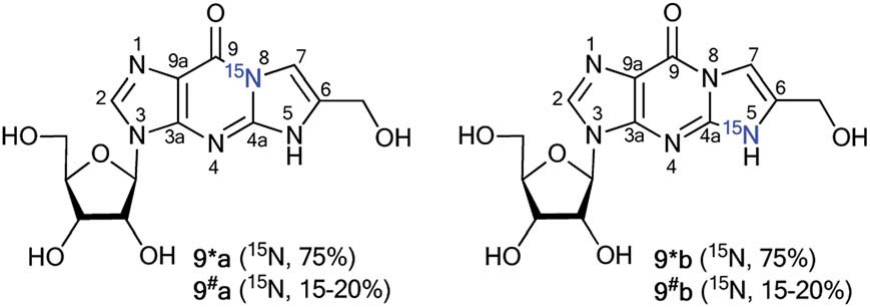
Alternative isomers 9*a/9^#^a and 9*b/9^#^b.

The values of ^1^*J*_C7–N8_ and ^1^*J*_C6–N5_ were obtained from the carbon spectra of compounds 8*a and 8*b with 75% ^15^N enrichment. This characteristic permitted the determination of sites for the attachment of the hydroxymethyl group in compounds 8*a and 8*b. However, the alternative isomers 9*a/9^#^a and 9*b/9^#^b were not observed (Fig. 1).

**Scheme 5:**
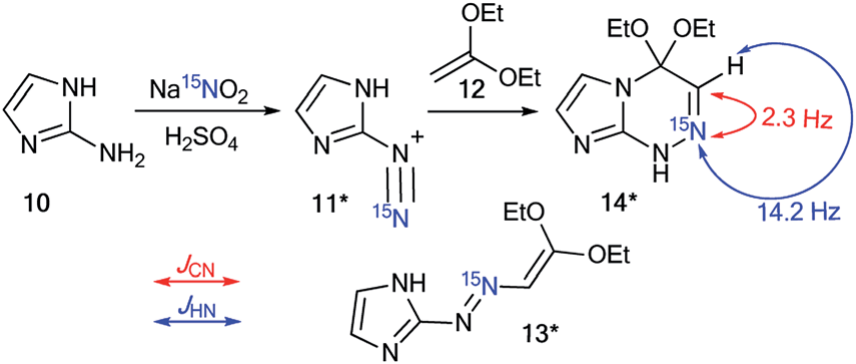
The choice between open chain azoalkene 13* and fused azoloazine 14*. The observed ^1^*J*_CN_ and ^2^*J*_HN_ couplings are shown by red and blue arrows, respectively.

The values of the ^1^H–^15^N and ^13^C–^15^N couplings can be applied as diagnostic features for confirmation of the ring closure of heterocycles. This approach was described in 1986 by Villarasa *et al.*, where a ^15^N-labeled derivative of diazoazole 11* was prepared.^33^ The synthesis comprises the diazotation of 2-aminoimidazole 10 with Na^15^NO_2_ having 25% ^15^N enrichment (Scheme 5). The following reaction between compound 11* and 1,1-dimethoxyethene 12 can lead to either open-chain azoalkene 13* or fused azoloazine 14*. It should be noted that compound 14* is the product of the cyclization of 13*. The observed ^1^*J*_CN_ of 2.3 Hz and ^2^*J*_HN_ of 14.2 Hz for the obtained compound were in good agreement with the NMR spectral data of pyridine and other azines. Thus, they confirmed the formation of bicycle 14* in the reaction of imidazole 11* with dimethoxyethene compound 12.

The use of labeled azole derivatives in the synthesis of fused heterocycles is one method for the incorporation of ^15^N atoms in the azoloazine series. Obtaining ^15^N-azoles can require several steps. For example, the synthesis of [2-^15^N]-5-amino-1,2,4-triazole 18* began by the interaction between labeled potassium nitrate (87%, ^15^N) and guanidine sulfate 15 (Scheme 5).^34^ Then, the N-nitrated product 16* was reduced to ^15^N-aminoguanidine bicarbonate 17*, which was transformed into [1-^15^N]-3-amino-1,2,4-triazole 18* by reaction with formic acid.

**Scheme 6:**
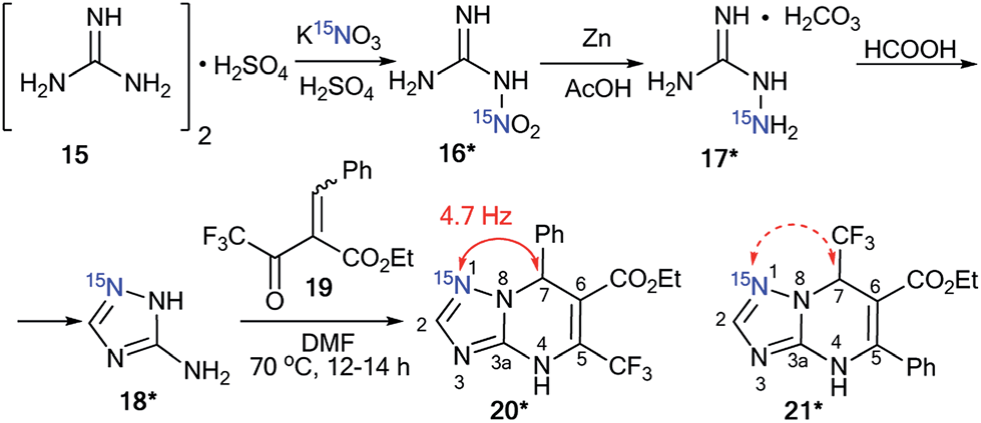
Structural elucidation of a series of 1,2,4-triazolo[1,5-*a*]pyrimidines. The observed ^2^*J*_CN_ coupling is shown by a red arrow. The expected coupling (^2^*J*_CN_) in the excluded structure is indicated by a dashed arrow.

Compound 18* (∼87%, ^15^N) with one equivalent of unlabeled 3-amino-1,2,4-triazole was used in a condensation reaction with 2-benzylidene-2-fluoroacyl ester 19 (Scheme 6).^35^ The analysis of the *J*_CN_ couplings showed that a ^13^C–^15^N spin–spin interaction was observed for the C7 atom bonded to a phenyl fragment (^2^*J*_CN_ = 4.7 Hz). Moreover, a ^2^*J*_H2–N1_ of 15.3 Hz was detected in the ^1^H NMR spectrum of compound 20*. These data unequivocally prove the formation of structure 20* (∼43%, ^15^N). In the case of obtaining the alternative product 21*, ^13^C–^15^N splitting should be observed for the signal of the carbon atom coupled with a CF_3_ group.

**Scheme 7:**
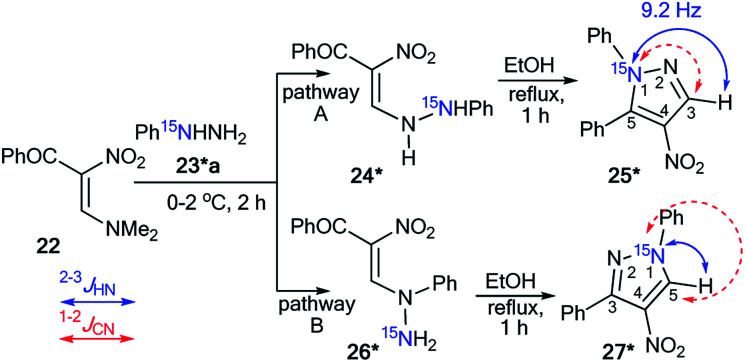
Establishing the structure of isomeric nitropyrazole 25*. The observed *J*_HN_ couplings are shown by blue arrows. The expected but unobserved *J*_CN_ are couplings indicated by dashed red arrows.

The use of ^15^N-phenylhydrazine 23*a in the interaction with nitroenamine 22 allowed for the determination of a reaction pathway that can be route A or B (Scheme 7).^36^ According to pathway A, product 25* is formed through 24*, while the alternative pathway B involves the formation of compound 26* that transforms into resulting pyrazole 27*. The comparison of the detected *J*_HN_ of 9.2 Hz with the early described amplitudes of the ^1^H–^15^N SSCCs for the pyrazole series showed that the observed coupling is a vicinal one. Thus, it was found that the reaction between 22 and 23*a gave compound 25*. Moreover, the formation of azole 27* would lead to the appearance of ^1^*J*_C5–N1_ with values of 8–11 Hz, whereas a carbon coupled with a hydrogen did not show the splitting of ^13^C–^15^N due to the small amplitude of ^2^*J*_C3–N1_ (>2 Hz). The weak ^13^C3–^15^N1 constant was used as an additional criterion for the confirmation of the structure of 25*.

### 2.2. Study of ring-chain tautomerism using *J*_CN_ and *J*_HN_ coupling constants

**Scheme 8:**
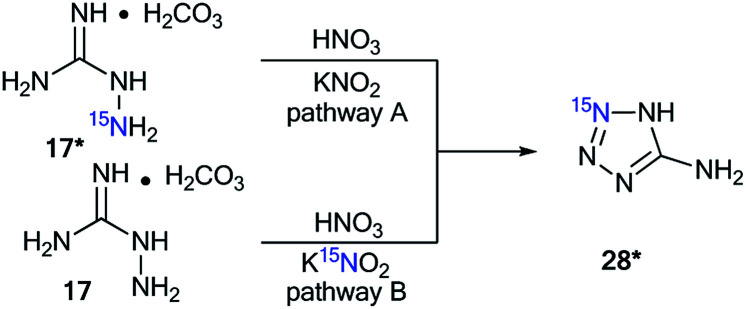
Synthesis of ^15^N-labeled aminotetrazole 28*.

Aminoguanidine bicarbonate 17* (^15^N, 86%) was also applied as a labeled starting material for the incorporation of ^15^N atoms in aminotetrazole 28* (pathway A, Scheme 8). The synthesis of 28* was based on the Thiele method,^37^ which includes the interaction of aminoguanidine salts with nitrous acid produced *in situ* from potassium nitrite and nitric acid.

The reaction of ^15^N-potassium nitrite (^15^N, 86%) with unlabeled aminoguanidine 17 is an alternative method that is suitable for obtaining compound 28* (pathway B, Scheme 8).

**Scheme 9:**
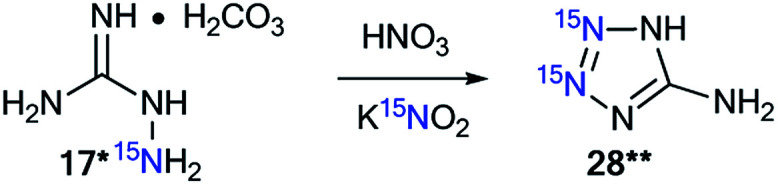
Synthesis of ^15^N double-labeled aminotetrazole 28**.

The use of ^15^N-aminoguanidine bicarbonate 17* and ^15^N-potassium nitrite allowed for the incorporation of two stable isotopes into aminotetrazole 28** (Scheme 9).^38^

**Scheme 10:**
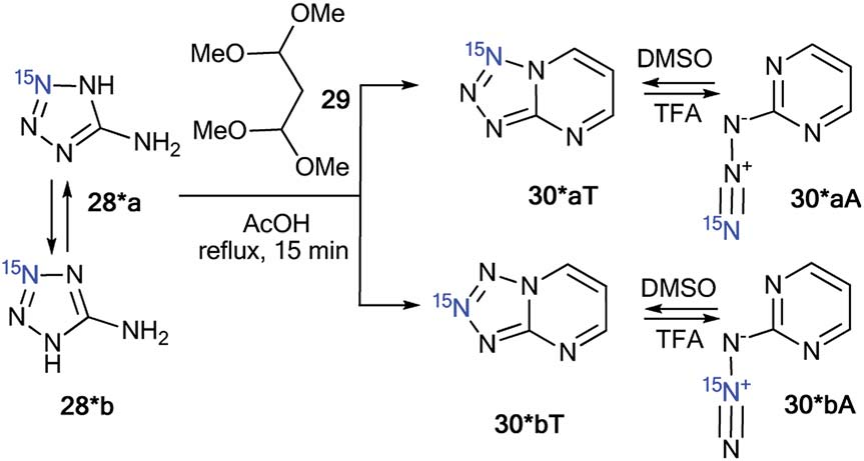
The formation of two isotopomeric ^15^N-labeled azides 30*A and tetrazoles 30*T in the reaction of aminotetrazole 28*.

Compounds 28* and 28** were used for the synthesis of labeled tetrazolo[1,5-*a*]pyrimidines and tetrazolo[1,5-*b*][1,2,4]triazines. Because ^15^N-aminotetrazole 28* exists in two tautomeric forms in solution, 28*a and 28*b, the reaction of this compound should result in the formation of a mixture of isotopomers (Scheme 10).^39^ As expected, the condensation of tetrazole 28* with 1,1,3,3-tetramethoxypropane 29 led to a mixture of tetrazolopyrimidines 30*aT and 30*bT in a 1 : 1 ratio.

**Scheme 11:**
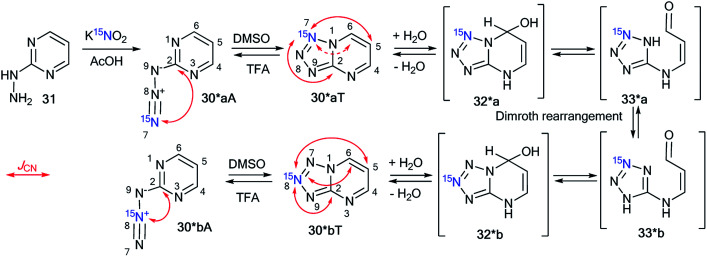
The Dimroth rearrangement of tetrazolopyrimidines 30*aT and 30*bT. The azide–tetrazole equilibrium is shifted to the tetrazole/azide form in DMSO-d_6_/TFA-d solution. The observed *J*_CN_ couplings from the ^15^N7 and ^15^N8 nuclei are shown by red arrows. The expected coupling (^2^*J*_CN_) is indicated by a dashed arrow. The measured *J*_CN_ values are presented in Tables 1 and 2.

A similar result was obtained by the reaction of 2-hydrazinopyrimidine 31 with ^15^N-enriched nitrous acid that was generated from labeled potassium nitrite in acetic acid (Scheme 11).^37^ A mixture of isotopomers 30*aT and 30*bT in a 5 : 1 ratio was obtained. The Dimroth rearrangement is one of the most likely sources of the observed isomerization.

The NMR spectra of the isotopomer mixture in DMSO-d_6_ solution showed that the tetrazole forms 30*a,bT prevail over the azides 30*a,bA, which were detected in trace amounts (∼5%). The detection of *J*_CN_ couplings for the C2, C5, and C6 nuclei (Scheme 11 and Table 1) proved the formation of a fused tetrazole (Scheme 10). Interestingly, small amounts of the azide forms 30*a,bA (∼5%) were detected in both the ^13^C and ^1^H NMR spectra. Similarly, *J*_CN_ splitting, which was only observed for the C2 nucleus, confirmed the azide structure of 30*a,bA. In TFA solution, the azide–tetrazole equilibrium was rapidly shifted toward the azides 30*a,bA. As expected for the azide forms 30*a,bA, the presence of ^13^C–^15^N *J*-couplings was detected only for the C2 nucleus (Scheme 11 and Table 2).

**Table 1:** *J*_CN_ couplings (Hz) of tetrazolo[1,5-*a*]pyrimidines and tetrazolo[1,5-*b*][1,2,4]triazines^a^

Compound	Solvent	*J*_C2–N7_	*J*_C2–N8_	*J*_C5–N7_/*J*_C6–N7_	*J*_C5–N8_/*J*_C6–N8_
30*a,bT^b^	DMSO-d_6_	2.3	2.5	1.0/3.8^c^	0.5/<0.3^d^
37*a,bT	DMSO-d_6_	3.3	2.1	1.6	0.9
37*a,bT	TFA-d	ND^e^	ND^e^	1.6	0.7
37**T^c^	DMSO-d_6_	3.3	2.0	1.5	0.8
40*a,bT	DMSO-d_6_	3.0	2.2	1.1	1.0
40*a,bT^f^	TFA-d	∼0.5	∼0.5	∼1.0	∼1.0
44*T	DMSO-d_6_	3.1		1.4	
44*T	TFA-d	<0.3^e^		1.7	

a Unless otherwise stated, the listed *J*_CN_ values represent the average between two independent measurements using ^13^C line-shape analysis and amplitude-modulated spin-echo experiments.

b The mixture of isotopomers 30*a/30*b (5 : 1) synthesized by Scheme 11 was used for the *J*_CN_ measurements.

c The *J*_CN_ value was measured only by ^13^C line-shape analysis.

d The *J*_CN_ coupling constant was not detected, probably due to the low abundance of the 30*b isotopomer.

e Measurements of the *J*_CN_ couplings were impossible due to the significant broadening of the corresponding ^13^C resonance.

f The *J*_CN_ values were estimated from the ^13^C line-shape analysis. Precise measurements were impossible due to the fast conversion of tetrazoles 40*a,bT to azides 40*a,bA in TFA solution.

**Table 2:** *J*_CN_ couplings (Hz) of azidopyrimidines and azido-1,2,4-triazines^a^

Compound	Solvent	*J*_C2–N7_	*J*_C2–N8_
30*a,bA^b^	DMSO-d_6_	0.6^c^	ND^d^
30*a,bA^b^	TFA-d	0.5	0.7
37*a,bA	DMSO-d_6_	ND^e^	ND^e^
37*a,bA	TFA-d	0.5	0.8
40*a,bA	DMSO-d_6_	ND^f^	ND^f^
40*a,bA	TFA-d	0.5	0.8
44*A	TFA-d	0.5	

a Unless otherwise stated, the listed *J*_CN_ values represent the average between two independent measurements using ^13^C line-shape analysis and amplitude-modulated spin-echo experiments.

b The mixture of isotopomers 30*a/30*b (5 : 1) synthesized by Scheme 11 was used for the *J*_CN_ measurements.

c The *J*_CN_ value was measured only by ^13^C line-shape analysis.

d The measurement of the *J*_CN_ coupling was impossible due to the low abundance of the 30*b isotopomer.

e Signals from the azide forms 37*a,bA were not observed in DMSO-d_6_ solution.

f The measurement of *J*_CN_ was impossible due to the low concentration of the azide forms 40*a,bA in DMSO-d_6_ solution.

**Scheme 12:**
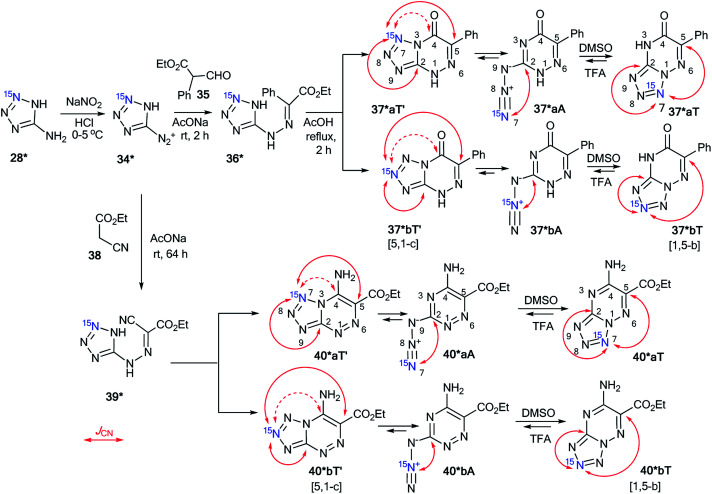
The azide–tetrazole equilibrium in the series of azido-1,2,4-triazines. The azide–tetrazole equilibrium is shifted to the tetrazole/azide form in DMSO-d_6_/TFA-d solution. The observed *J*_CN_ couplings from the ^15^N7 and ^15^N8 nuclei are shown in red. The expected but unobserved couplings (^2^*J*_CN_ and ^3^*J*_CN_) are indicated by dashed arrows. The measured *J*_CN_ values are presented in Tables 1 and 2.

The cyclization of the diazonium salt 34*, derived from ^15^N-labeled aminotetrazole 28*, with ethyl α-formylphenylacetate 35 gave a mixture of tetrazolo[1,5-*b*][1,2,4]triazines 37*aT and 37*bT at a 1 : 1 ratio (Scheme 12).^37^ Similarly, a 1 : 1 isotopomeric mixture of tetrazolo[1,5-*b*][1,2,4]triazines 40*aT and 40*bT was obtained by the reaction of 34* with ethyl cyanoacetate 38 (Scheme 12).

Compounds 37*a,bT were only registered in the tetrazole form in DMSO-d_6_. The observation of the ^13^C–^15^N splitting for the C2 and C5 carbon signals in the 1D ^13^C NMR spectra of 37*a,bT unambiguously confirmed the [1,5-*b*] fusion between the tetrazole and 1,2,4-triazine rings.

A small amount of the azide forms 40*a,bA (∼4%) in DMSO-d_6_ solution was detected in the ^13^C and ^1^H NMR spectra for the sample that was obtained by the interaction of compounds 34* and 38 (Table 2). The low concentration of the azide form did not allow for the detection or measurement of the corresponding *J*_CN_ couplings. This form was only characterized by a relatively large downfield shift of the C2 resonance. Moreover, the main tetrazole isomers 40*a,bT were characterized by *J*_CN_ couplings for signals C2 and C5. These characteristics allowed for the determination of the type of fusion between the tetrazole and 1,2,4-triazine rings in heterocycles 40*a,bT as [1,5-*b*].

The NMR spectral parameters of compounds 37*a,bT were determined in TFA immediately after dissolving the tetrazole form and 30 days after the preparation of the solution. During this period of time, the relative population of the azide forms of 37*a,bA increased from 0% to 60%. Significant broadening of the C2 signal observed in the ^13^C spectra of 37*a,bT in TFA did not allow for the identification of the *J*_CN_ couplings from this carbon atom (Table 1). The ^3^*J*_C5–N7_ and ^4^*J*_C5–N8_ values for the C5 nucleus were measured easily. The obtained values of 1.6 and 0.7 Hz, respectively, correspond nicely to the *J*-couplings observed for the C5 carbon of 37*a,bT in DMSO-d_6_ solution (1.6 and 0.9 Hz, respectively). This similarity confirms the retention of the bicyclic structure with a [1,5-*b*] type of fusion between the azole and azine fragments for 37*a,bT in TFA (Scheme 12). An analysis of the ^13^C multiplets of 37*a,bA in the 1D NMR spectra revealed the presence of *J*_CN_ interactions only for the C2 nucleus, which confirmed the azide structure of this compound (Table 2 and Scheme 12).

The rearrangement of 40*a,bT to 40*a,bA in TFA solution was relatively fast when compared to that of compounds 40*a,bT. The ^13^C NMR spectra were measured 1, 2 and 12 h after dissolving 40*a,bT in TFA, and the concentrations of the azide forms 40*a,bA were 18%, 40% and 97%, respectively. The *J*_CN_ interactions for the C2 and C5 carbon atoms of tetrazole 40*a,bT were observed in the ^13^C NMR spectra (Scheme 12 and Table 1), thus proving that the tetrazole structure was retained and that the [1,5-*b*] type of fusion for 40*a,bT occurred. The precise measurement of the *J*_CN_ values for 40*a,bT was impossible due to the fast conversion of tetrazoles to azides. A simplified line-shape analysis, however, revealed that the ^2^*J*_C2–N7_ and ^2^*J*_C2–N8_ interactions in 40*a,bT have significantly lower values in TFA than in DMSO-d_6_ (Table 1). The azides 40*a,bA in TFA solution were characterized by the presence of ^13^C–^15^N *J*-coupling constants for only the C2 carbon (Scheme 12 and Table 2).

**Scheme 13:**
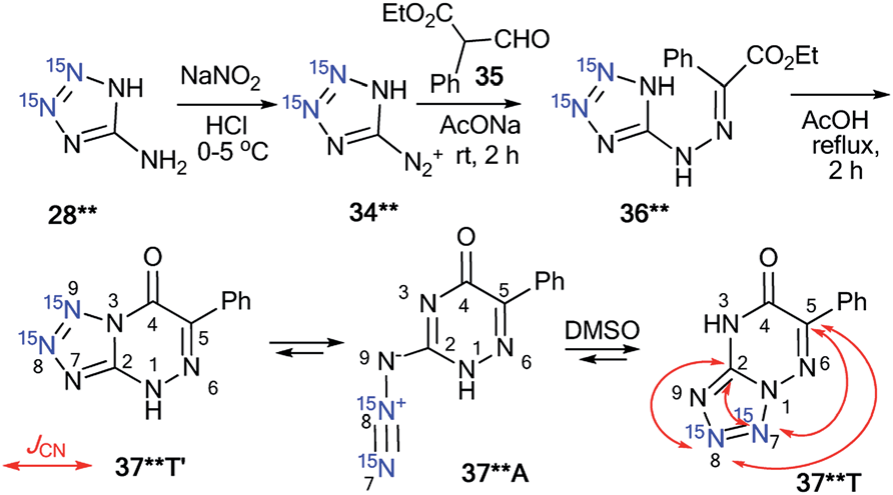
The azide–tetrazole equilibrium in double-labeled 1,2,4-triazine 37**. The observed ^13^C–^15^N coupling constants in DMSO-d_6_ solution are shown by red arrows.

The use of double-labeled aminotriazole 28** is one way to avoid the formation of isotopomeric mixtures. For example, the reaction of diazonium salt 34**, obtained from 28**, with ethyl α-formylphenylacetate 35 gave compound 36**, which underwent cyclization in acetic acid (Scheme 13).^39^ As a result, ^15^N_2_-tetrazolo[1,5-*b*][1,2,4]triazine 37**T was synthesized. An analysis of the *J*_CN_ couplings confirmed the type of fusion between the tetrazole and 1,2,4-triazine rings in compound 37**T (Table 1 and Scheme 13).

**Scheme 14:**
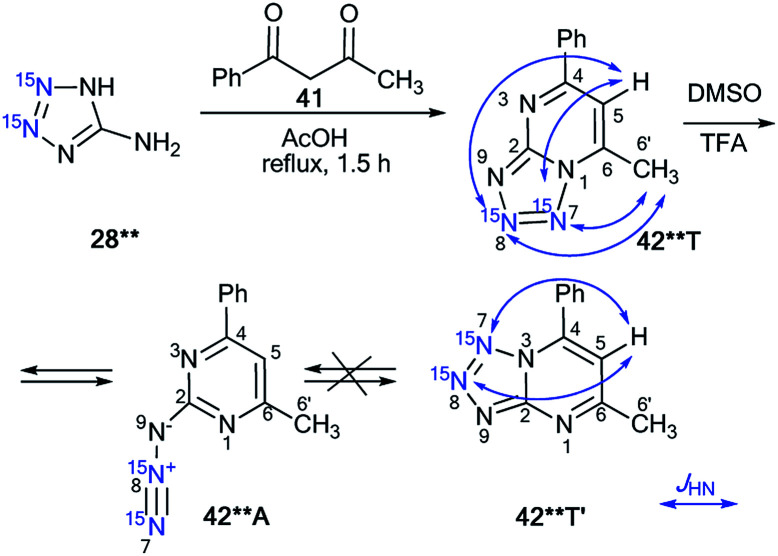
The azide–tetrazole equilibrium in double-labeled pyrimidine 42**. The observed long-range ^1^H–^15^N7 *J* coupling constants (^4–5^*J*_H6′/H5–N7/N8_) are shown by blue arrows. The measured *J*_HN_ values are presented in Table 3.

A similar approach was applied for the incorporation of two ^15^N atoms in the structure of tetrazolo[1,5-*a*]pyrimidine derivatives. The interaction of 28** with benzoylacetone 41 led to product 42**T (Scheme 14).^38^ The structure of compound 42**T in DMSO-d_6_ was determined by the analysis of the long-range ^1^H–^15^N coupling constants, which were measured by spin-echo experiments with the selective inversion of ^15^N nuclei. The observed ^*n*^*J*_HN_ values are presented in Table 3. The ^4^*J*_H6′–N7_ and ^5^*J*_H6′–N8_ couplings can only be observed in the proton spectrum of compound 42**T (Scheme 14). In the case of the formation of tetrazoloazine 42**T′, these spin–spin interactions should be absent.

**Table 3:** Long-range *J*_HN_ couplings (Hz) of tetrazolo[1,5-*a*]pyrimidines and tetrazolo-1,2,4-triazines^a^

Compound	Solvent	*J*_H5–N7_/*J*_H10–N7_	*J*_H5–N8_/*J*_H11–N7_	*J*_H6′–N7_/*J*_H12–N7_	*J*_H6′–N8_/*J*_H13–N7_
42**T	DMSO-d_6_	0.20	0.82	0.09	0.08
46*T	DMSO-d_6_/TFA-d (3/1)	0.06	0.06		
46*T′	DMSO-d_6_	0.11	0.04	0.04	0.15
46*T′	DMSO-d_6_/TFA-d (3/1)	0.11	0.04	0.04	0.16
46*T′	TFA-d	0.13	ND^b^	ND^b^	0.17
48*T	DMSO-d_6_	0.6		0.2	
48*T	DMSO-d_6_/TFA-d (2/3)	0.6		0.2	
48*T	TFA-d	0.7		0.2	
48*T′	DMSO-d_6_	0.6			
48*T′	DMSO-d_6_/TFA-d (2/3)	0.6			

a The ^1^H–^15^N7 *J* coupling constants (*J*_HN_) were measured by amplitude modulated spin-echo experiments.

b Not determined due to overlap.

This result indicated that the analysis of ^4^*J*_HN_ and ^5^*J*_HN_ can be used for the determination of the method of fusion between azole and azine fragments in tetrazolo[1,5-*a*]pyrimidines.

It was also found that tetrazole isomer 42**T underwent complete rearrangement into the azide form 42**A in TFA solution. This was confirmed by the disappearance of the long-range ^1^H–^15^N7/8 correlations in the proton spectrum.

**Scheme 15:**
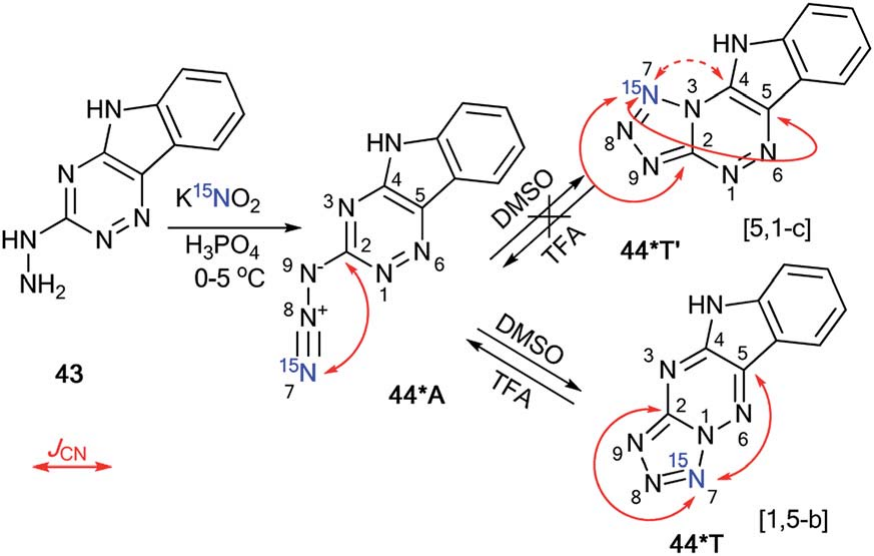
The azide–tetrazole equilibrium of 1,2,4-triazine 44*. The azide–tetrazole equilibrium is shifted to the tetrazole/azide form in DMSO-d_6_/TFA-d solution. The observed *J*_CN_ couplings from the ^15^N7 nucleus are shown in red. The expected but unobserved coupling (^2^*J*_CN_) is indicated by a dashed arrow. The measured *J*_CN_ values are presented in Tables 1 and 2.

In contrast to 2-hydrazinopyrimidine 31, the reaction of compound 43 with ^15^N-potassium nitrite (^15^N, 86%) in phosphoric acid led to the single isotopomer 44*T (Scheme 15).^37^ Registration of the 1D ^13^C NMR spectra of 44*T in DMSO-d_6_ showed *J*_CN_ splittings for the signals from the C2 and C5 nuclei (Scheme 15 and Table 1).

These spin–spin interactions confirmed the formation of a cyclic structure with a [1,5-*b*] type of fusion between the tetrazole and 1,2,4-triazine fragments. If the alternative isomer 44*T′ was present, then additional *J*_CN_ splitting for the C4 signal should be observed in the carbon spectrum (Scheme 15). The azide–tetrazole equilibrium was shifted to the azide form 44*A in TFA solution. The rearrangement of tetrazole 44*T to the azide did not occur quickly.

The NMR spectra for compound 44*T were measured in TFA-d solution immediately after dissolving the tetrazole form, after 16 days, and again after 30 days. During this period, the relative population of isomer 44*A increased gradually from 0% to 87%. An analysis of the *J*_CN_ couplings allowed for the detection of both 44*T and 44*A in TFA-d (Tables 1 and 2).

**Scheme 16:**
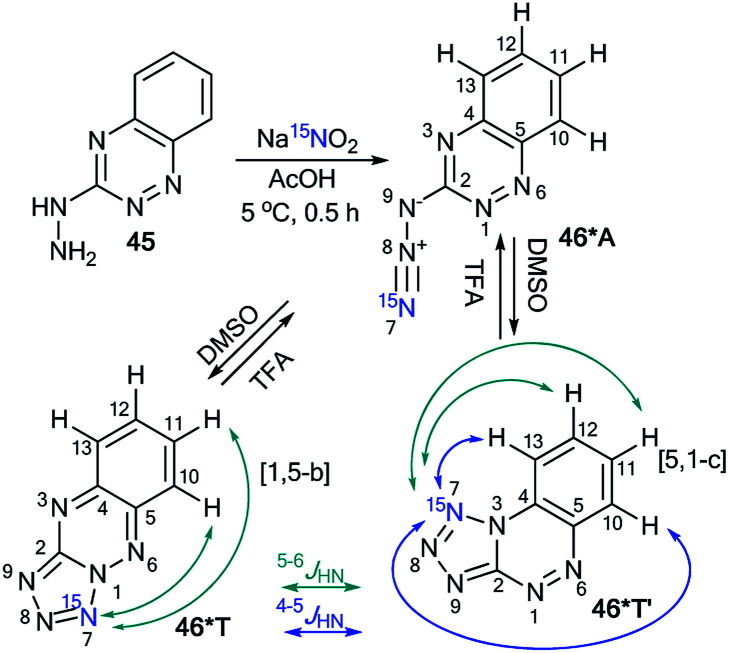
The azide–tetrazole equilibrium of azido-1,2,4-triazine 46*. The observed ^1^H–^15^N7 *J* coupling constants (*J*_HN_) with magnitudes of *J* ≥ 0.1 Hz and *J* < 0.1 Hz are indicated by the blue and green arrows, respectively.

**Scheme 17:**
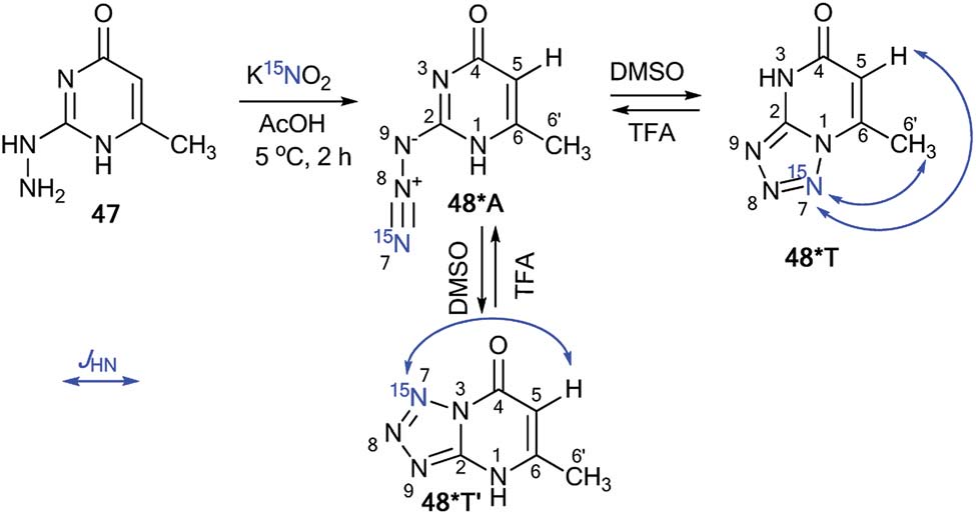
The azide–tetrazole equilibrium in pyrimidine 48*. The observed ^1^H–^15^N7 *J* coupling constants are indicated by the blue arrows.

Similar to compound 43, the treatment of hetarylhydrazines 45 and 47 with labeled sodium nitrite (^15^N, 98%) in acetic acid selectively yielded compounds 46*A and 48*A, respectively (Schemes 16 and 17).^40^ The selective incorporation of a ^15^N label into the azolo core of the tetrazoloazine leads to the appearance of long-range ^1^H–^15^N *J* couplings, which can be easily measured in a quantitative fashion using 1D spin-echo experiments.

Compound 46* in DMSO-d_6_ underwent a transformation to an isomeric mixture of 1,2,4-triazine derivatives 46*T′, 46*A and 46*T in a ratio of 79 : 12 : 9, respectively. The addition of TFA-d to DMSO-d_6_ solution led to an increase in the relative concentration of the minor forms A and T.

In pure TFA, compound 46* underwent an almost complete rearrangement to yield the open-chain azide 46*A. In this case, the minor isomer 46*T′ was also found with a concentration of approximately 1%. The measurement of ^4–6^*J*_HN_ for tetrazole 46*T′ was possible in DMSO-d_6_, TFA-d and different mixtures of these solvents (Table 3). The detection of ^15^N7–^1^H13 and ^15^N7–^1^H12 spin–spin interactions provides evidence for the [5,1-*c*] type of fusion between the azole and azine rings in 46*T′. It should be noted that other protons signals (H11 and H12) can have *J*_H–N7_ couplings. The disappearance of the *J*_H13–N7_ and *J*_H12–N7_ couplings and the measurement of the values of *J*_H10–H7_ and *J*_H11–N7_ allowed the confirmation of the structure of 46*T (Table 3 and Scheme 16).

Two tetrazole forms, 48*T′ and 48*T, in a ratio of 1 : 1 were detected upon dissolution of 48*A in DMSO-d_6_. The signals from azide 48*A in the NMR spectra became detectable only in the mixture DMSO-d_6_/TFA-d at a concentration of the acid of more than 50%. For example, in DMSO-d_6_/TFA-d (3 : 1) solution, a 24 : 29 : 47 concentration ratio of 48*T′ : 48*A : 48*T was found. In TFA-d, a mixture of the isomers 48*A and 48T (96 : 4) was obtained. The observed *J*_H5–N7_ and *J*_H6′–N7_ patterns enable the unambiguous determination of the cyclization method of the azole fragment in tetrazolopyrimidines 48*T′ and 48*T in DMSO-d_6_, TFA-d and a mixture of DMSO-d_6_/TFA-d (Table 3 and Scheme 17).

**Scheme 18:**
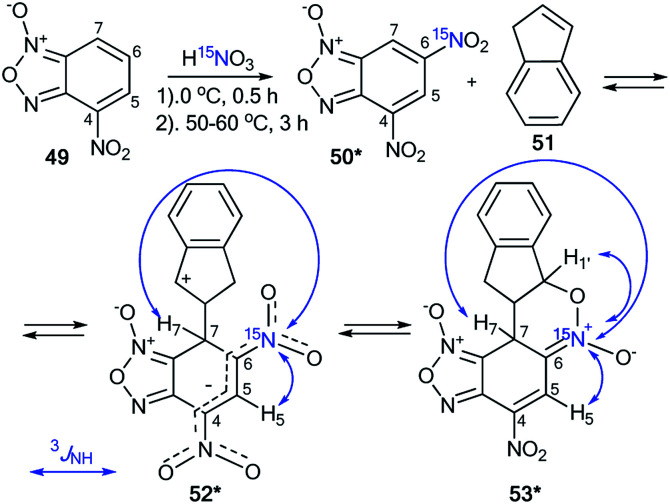
The study of the interaction between dinitrofurazone 50* and indene using a ^15^N label. The observed ^1^H–^15^N6 *J* coupling constants are indicated by the blue arrows.

The treatment of nitrobenzofuroxan 49 with ^15^N-nitric acid (^15^N, 98%) gave enriched compound 50* (Scheme 18).^41^ The incorporation of the isotope label in the 6-position of dinitrobenzofuroxan 50* permitted the determination of the specificity of the reaction with indene 51. The appearance of ^3^*J*_H5–N6_ = 1.9 Hz, ^3^*J*_H7–N6_ = 3.1 Hz and ^3^*J*_H1′–N6_ = 7.1 Hz in the proton spectrum showed that the expected σ-adduct 52* underwent a transformation into N-oxide 53*. If the isomerization of 52* into 53* had not occurred, the ^3^*J*_H1′–N6_ coupling would have been absent.

**Scheme 19:**
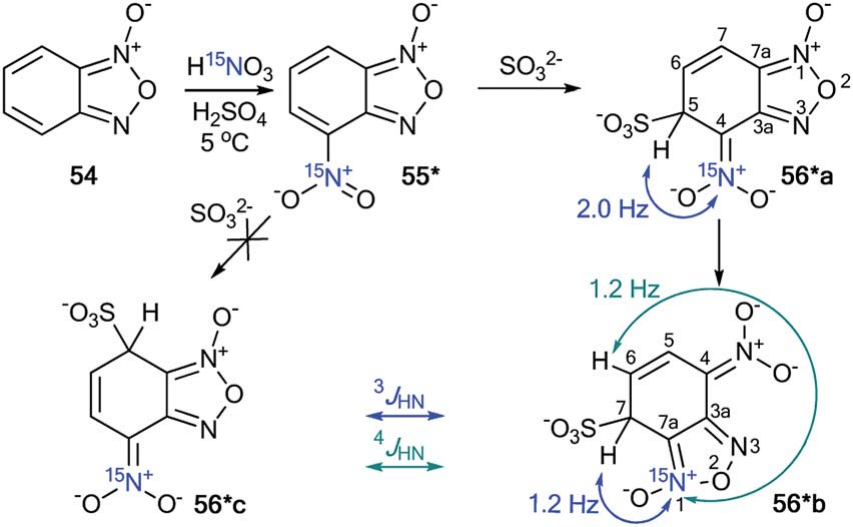
The study of the interaction between nitrofurazone 55* and SO_3_^2−^ using a ^15^N label. The observed ^3^*J*_HN_ and ^4^*J*_HN_ couplings are shown by blue and green arrows, respectively.

The incorporation of the ^15^N isotope in structure 55* was achieved by the nitration of compound 54 with labeled nitric acid (Scheme 19).^42^ The appearance of the ^15^N atom provided the opportunity to study the interaction of 55* with sulfite ions (Scheme 19). It was found that the initial reaction occurs at the 5-position to give the σ-adduct 56*a, which was characterized by a geminal ^1^H–^15^N coupling constant of 2.0 Hz. Then, isomerization of intermediate 56*a occurred to yield adduct 56*b carrying a sulfite at the 7-position. ^4^*J*_H7–15N_ = 1.2 Hz and ^3^*J*_H6–15N_ = 1.2 Hz were observed in the proton spectrum of compound 56*b. These ^1^H–^15^N coupling constants showed that the authors observed the intramolecular Boulton–Katritzky rearrangement rather than the SO_3_^2−^ transfer, which should lead to the formation of compound 56*c.

**Scheme 20:**
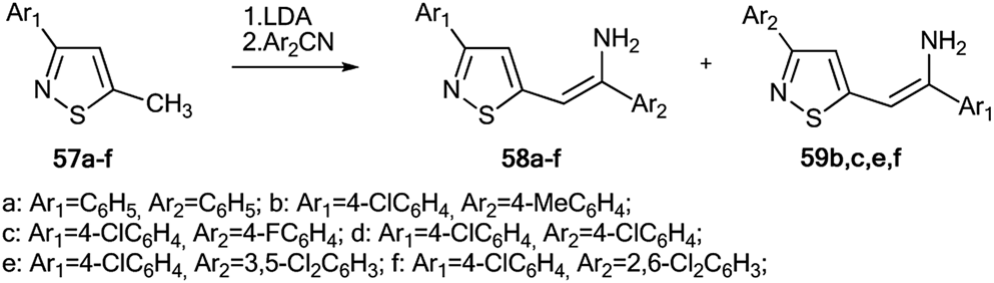
Reaction between 3-aryl-5-methylisothiazoles 57a–f and aromatic nitriles.

The use of ^15^N-labeled compounds allowed the examination of the interaction of 3-aryl-5-methylisothiazoles with 57a–f with aromatic nitriles in the presence of lithium diisopropylamide (LDA) (Scheme 20). The reaction between compounds 57b,c,e,f and nitriles with different aromatic fragments (Ar_1_ ≠ Ar_2_) afforded the formation of a mixture of isomers 58b,c,e,f and 59b,c,e,f. In the cases where Ar_1_ = Ar_2_, the reaction gave a single product.^43^ The application of ^15^N-enriched *p*-chlorobenzonitrile in these experiments allowed the authors to study the structures of the obtained compounds and the mechanism of formation of these products.

**Scheme 21:**
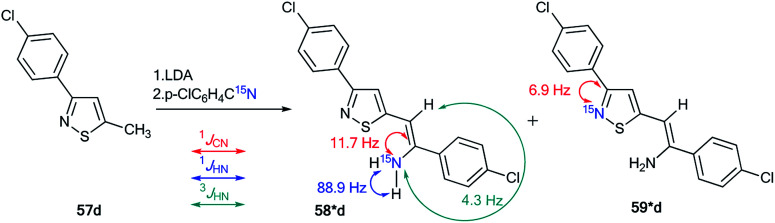
Evidence of the formation of two isomeric 3-arylisothiazoles 58*d and 59*d. The observed ^1^*J*_CN_, ^1^*J*_HN_ and ^3^*J*_HN_ couplings are shown by red, blue and green arrows, respectively.

The treatment of 3-(4-chlorophenyl)-5-methylisothiazole with LDA and ^15^N-labeled *p*-chlorobenzonitrile afforded a mixture of isotopomers 58*d and 59*d containing ^15^N atoms in the amino group and thiazole fragment, respectively (Scheme 21). The positions of the isotope label in heterocycles 58*d and 59*d were provided by the measurement of the corresponding direct ^13^C–^15^N coupling constants (11.7 Hz and 6.9 Hz for 58*d and 59*d, respectively). The value of ^3^*J*_HN_ = 4.3 Hz for the vinyl proton in isotopomer 58*d showed that the obtained compound had a *trans* geometry with respect to the two aromatic rings Ar_1_ and Ar_2_.

For an explanation of the formation of the isotopomeric mixture 58*d and 59*d, the selectively labeled compound 58*d was prepared, and the ring-chain transformation for this labeled structure was studied by NMR. Compound 58*d was obtained by treatment of 60 with ^15^N-labeled *p*-chlorobenzonitrile.

**Scheme 22:**
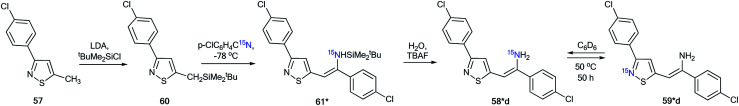
Selective synthesis of 3-arylisothiazole 58* and ^15^N label redistribution.

This procedure led to compound 61*, which then underwent desilylation to form isotopomer 58*d (Scheme 22). Compound 60 was obtained by the interaction of methylisothiazole with *tert*-butyldimethylsilyl chloride in the presence of LDA. Then, heating the C_6_D_6_ solution of 58*d at 50 °C for 50 h resulted in an equilibrium of a 1 : 1 mixture of isotopomers 58*d and 59*d.

**Scheme 23:**
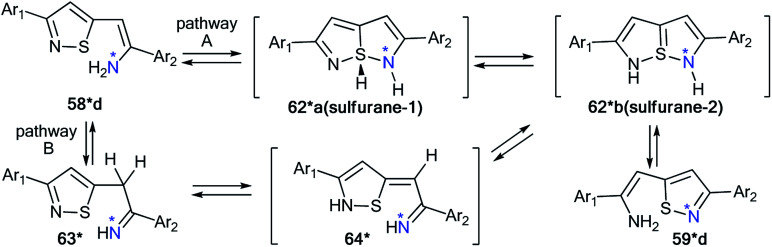
The possible mechanism of the ^15^N label redistribution in a series of isotope-enriched 3-aryl-5-enaminoisothiazoles.

Hence, two possible mechanisms (A and B) for the reversible equilibrium between 58*d and 59*d were presented (Scheme 23). It should be noted that the key intermediate in each path is sulfurane-2, 62*b. Pathway A involved the formation of sulfurane-1, 62*a, which transformed into structure 62*b*via* a [1,5]-sigmatropic hydrogen shift. Alternative pathway B suggests that thiazoles 63* and 64* are involved in the isomerization process.

Thus, the incorporation of ^15^N labels and the analysis of the *J*_CN_ and *J*_HN_ couplings allow the determination of the formation mechanism of different heterocyclic systems and the observation of ring-chain tautomerism in a series of heterocycles.

## 3. Analysis of *J*_CN_ and *J*_HN_ couplings as a method for the study of ring-chain transformations under the action of nucleophilic and electrophilic reagents

Some reactions of nitrogen heterocycles are simple at first glance, but they can hide a complex and completely unobvious mechanism. Isotopic labels allow a look deep inside into the details of such reaction mechanisms. Several heterocycles undergo ring transformations under the action of nucleophilic reagents (ANRORC, Dimroth rearrangement, *etc.*). Another type of recyclization is associated with intramolecular attack by the electron-deficient terminal atoms of the open-chain form on the ring system heteroatoms bearing a lone pair of electrons. It should be noted that ring opening or recyclization processes in both cases can occur and lead to the formation of a new type of heterocyclic structure. Additionally, determination of the structure of the obtained product always remains an important issue in organic chemistry. Moreover, solution of this task can help provide evidence for the mechanisms of ring-chain transformations.

### 3.1. Study of ANRORC and Dimroth rearrangements and similar chemical transformations by analysis of *J*_CN_ and *J*_HN_ couplings

**Scheme 24:**
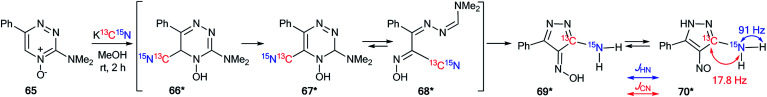
The structural determination of the interaction products of 1,2,4-triazine oxide with potassium cyanide using ^15^N and ^13^C labels. The observed ^13^C–^15^N and ^1^H–^15^N coupling constants are shown by red and blue arrows, respectively.

The analysis of *J*_CN_ and *J*_HN_ is an efficient method for studying different rearrangements that include ring-chain and ring opening transformations. It should be noted that the use of ^15^N labeling and the estimation of ^13^C–^15^N and ^1^H–^15^N coupling constants are a single approach for the determination of one-pot cascade reactions; for example, the study of the ANRORC mechanism in the reaction of N-oxide-1,2,4-triazine 65 with potassium cyanide (Scheme 24).^44^ The use of K^13^C^15^N showed that the product of nucleophilic addition 66* transformed into pyrazole 70* through the formation of intermediates 67*–69*. The structure of compound 70* was confirmed by the measurements of *J*_CN_ = 17.8 Hz and *J*_HN_ = 91 Hz. These characteristics show the unambiguous incorporation of the ^13^C atom in the pyrazole ring of product 70*.

**Scheme 25:**
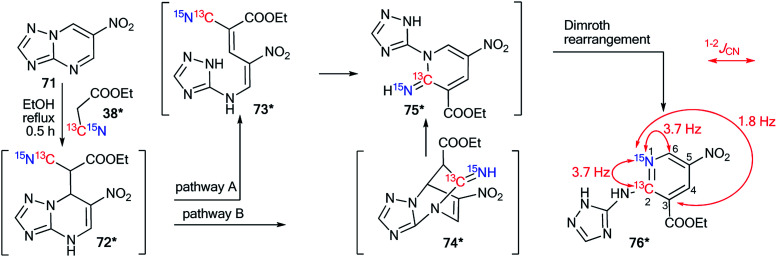
Establishment of the products and the mechanism of the reaction between nitrotriazolopyrimidine 71 and ethyl cyanoacetate. The observed ^13^C–^15^N coupling constants are shown by red arrows.

The application of double-labeled ^13^C, ^15^N-ethyl cyanoacetate 38* permitted the detection of the Dimroth rearrangement for nitro-1,2,4-triazolo[1,5-*a*]pyrimidine 71 (Scheme 25).^45,46^

It was found that ^13^C, ^15^N-ethyl cyanoacetate 38* demonstrated binucleophilic properties in the reaction with 6-nitro-1,2,4-triazolo[1,5-*a*]pyrimidine 71, and the mechanism of this reaction included several consecutive transformations (Scheme 25). The first step gave adduct 72*, which underwent isomerization into the open form 73* (pathway A) or structure 74* (pathway B). It should be noted that both intermediates 73* and 74* can transform into imine 75*. The following Dimroth rearrangement led to compound 76*. The usage of ^13^C- and ^15^N-labeled atoms and the analysis of ^13^C–^15^N coupling constants allowed for the detection of the C–^13^C–^15^N–C fragment in the pyridine ring of molecule 76*. This result was confirmed by the registration of ^13^C–^15^N splitting for signals C2 (^1^*J*_C–N1_ = 3.7 Hz), C6 (^1^*J*_C–N1_ = 3.7 Hz) and C3 (^2^*J*_C–N1_ = 1.8 Hz).

Recently, a representative study of a Dimroth-type ring-chain rearrangement in the imidazo[1,2-*a*]pyrimidine series using ^15^N-labeled samples and an analysis of ^1^H–^15^N couplings was described.^27^

**Scheme 26:**
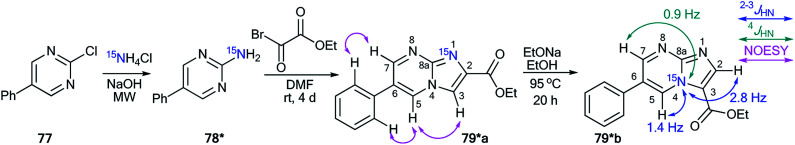
The Dimroth rearrangement in a series of imidazo[1,2-*a*]pyrimidines. The measured ^3^*J*_HN_ and ^4^*J*_HN_ couplings in the 1D ^1^H NMR spectrum of compound 79*b are shown by blue and green arrows, respectively. The observed ^1^H–^1^H NOE interactions between H-atoms in structure 79*a are shown by magenta arrows.

**Scheme 27:**
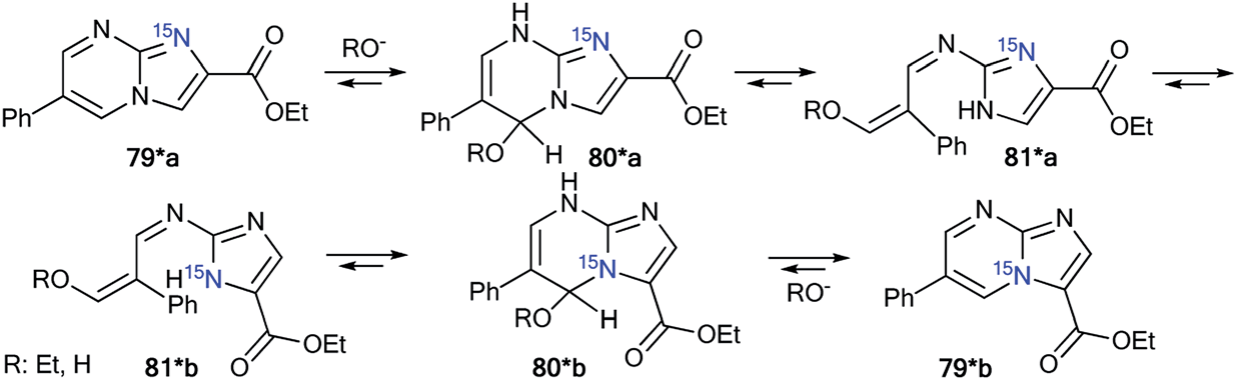
Mechanism of the Dimroth rearrangement of compound 79*a to isomer 79*b.

The incorporation of a ^15^N atom in molecule 79*a involved the substitution at C2 in compound 77 using ^15^NH_4_Cl under mildly basic conditions and cyclization of the obtained amine 78* with ethyl bromopyruvate (Scheme 26). The structure of imidazo[1,2-*a*]pyrimidine 79*a was determined by a 2D NOESY spectrum that showed a ^1^H–^1^H NOE interaction of the H5 atom with the phenyl *ortho* protons and proton H3 of the imidazole fragment. The formation of isomer 79*b was achieved by the treatment of compound 79*a with sodium ethylate. Overall, azoloazine 79*b containing a ^15^N label in position 4 of the imidazo[1,2-*a*]pyrimidine scaffold was obtained. The appearance of the labeled nitrogen atom at the bridgehead position in compound 79*b was confirmed by the additional ^1^H–^15^N4 splitting for the signals of H2, H5 and H7 (2.8 Hz, 1.4 Hz and 0.9 Hz, respectively) in the 1D ^1^H NMR spectrum. The values of the ^2^*J*_HN_, ^3^*J*_HN_ and ^4^*J*_HN_ couplings were in good agreement with the data from the 2D ^1^H–^15^N HMBC NMR experiment, which allowed the observation of same spin–spin interactions for heterocycle 79*b. Thus, it was shown that structure 79*a underwent a Dimroth rearrangement, which involves the addition of a hydroxide/alkoxide ion followed by ring opening (Scheme 27).

A similar result was obtained from the treatment of compound 79*a with a solution of sodium hydroxide in a mixture of EtOH and THF. In this case, the rearrangement was accompanied by hydrolysis of the ester group.

**Scheme 28:**
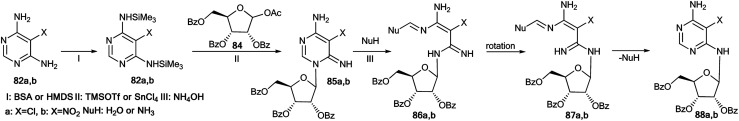
The Dimroth rearrangement under the action of nucleophiles in a pyrimidine series accompanied by ribose migration.

**Scheme 29:**
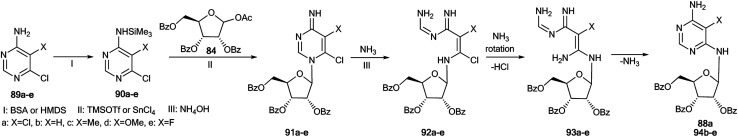
The Dimroth rearrangement under the action of ammonia in the pyrimidine series accompanied by ribose migration.

An unusual transformation was found from the study of Vorbrüggen glycosylation in a series of aminopyrimidines 82a,b and 89a–e using ^15^N-labeled regents (Schemes 28 and 29).^47^ It was revealed that the silylated 4-aminopyrimidines 83a,b or 90a–e after interaction with 84 yielded compounds 88a,b and 94b–e, which are products of the Dimroth rearrangement of nucleosides 85a,b and 91a–e in basic conditions. The mechanisms of these processes involve nucleophilic addition, ring opening and ring closure. Furthermore, the formation of structures 88a and 94a–e from 91a–e presumes the nucleophilic substitution of a chlorine atom in open intermediates 92a–e.

**Scheme 30:**
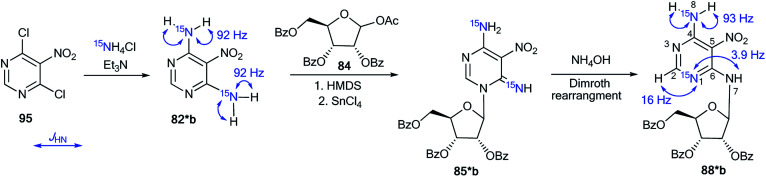
The Dimroth rearrangement in double-labeled nitrouridine. The observed ^1^H–^15^N coupling constants are shown by blue arrows.

**Scheme 31:**
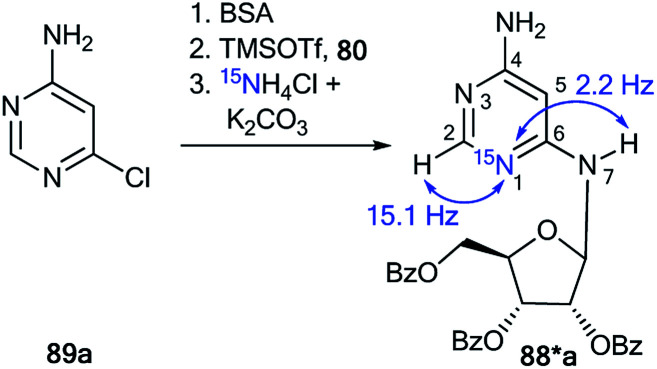
The ribosyl migration during the Dimroth rearrangement. The observed ^1^H–^15^N coupling constants are shown by blue arrows.

The synthesis of double-labeled diaminopyrimidine 82*b and the use of this compound in the glycosylation reaction led to product 88*b (Scheme 30). The observed ^1^*J*_H8–N8_ = 93 Hz, ^2^*J*_H2–N1_ = 16 Hz and ^3^*J*_H7–N1_ = 3.9 Hz unambiguously confirmed the structure of compound 88*b. These characteristics showed that the pyrimidine derivative 88*b was formed by the Dimroth rearrangement of the ribosylated azine 85*b. The mechanism presented in Scheme 29 was confirmed by the application of ^15^N-ammonium chloride in the process of the glycosylation of compound 89a (Scheme 31). In this case, the incorporation of an isotope label in the pyrimidine ring was also observed. The values of the ^1^H–^15^N coupling constants (^2^*J*_H2–15N_ = 15.1 Hz and ^3^*J*_H7–15N_ = 2.2 Hz) unequivocally proved the incorporation of a ^15^N atom into a pyrimidine fragment in compound 88*a.

**Scheme 32:**
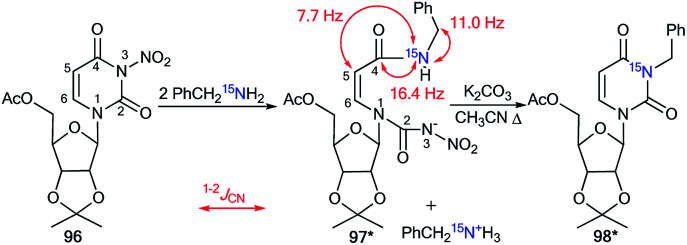
The incorporation of ^15^N atom into the structure of the uridine derivative. The observed ^1–2^*J*_CN_ couplings are shown by red arrows.

The application of ^15^N-benzylamine (prepared by the reduction of PhCO^15^NH_2_) in the reaction with *N*-nitrouridine 96 showed that the nucleophilic attack mainly occurs at the C4 atom of the uridine ring.^48^ Moreover, this interaction afforded ring-chain intermediate 97*, which transformed into compound 98* in the presence of K_2_CO_3_ (Scheme 32). The production of structure 97* was confirmed by analysis of the ^13^C–^15^N coupling constants. Indeed, the *J*_CN_ splittings were only detected for C4 (^1^*J*_CN_ = 16.4 Hz) and C5 (^2^*J*_CN_ = 7.7 Hz) atoms, which unambiguously proved the production of the ring opening product 97*. The formation of intermediate 97* was monitored by an NMR experiment in CDCl_3_ solution. This reaction is one example that allows the incorporation of a ^15^N-labeled atom in the core of a uridine derivative.

**Scheme 33:**
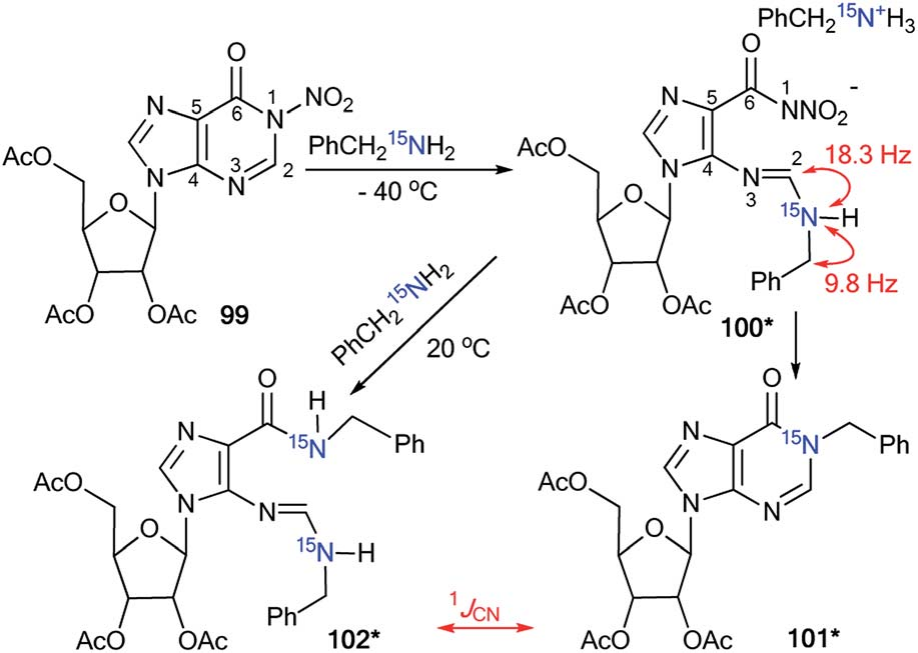
The incorporation of ^15^N atom into the structure of a hypoxanthine derivative. The observed ^1^*J*_CN_ couplings are shown by red arrows.

A previously mentioned article^48^ also describes the study of the features of the interaction of *N*-nitroinosine with an amine by using ^15^N-labeled benzylamine (Scheme 33). The reaction of 99 with PhCH_2_^15^NH_2_ was monitored by NMR in CDCl_3_ solution. It was shown that the attack of the amine takes place at the C2 atom of the inosine nitro derivative 99. This process leads to the ring opened intermediate 100*, which may follow two pathways, either 101*, or, in the presence of an excess of ^15^N-labeled benzylamine, compound 102*. In the 1D ^13^C NMR spectrum of 100*, the interaction ^13^C–^15^N was only observed for signal C2 (^1^*J*_CN_ = 18.3 Hz) and the carbon atom of the benzyl fragment (^1^*J*_CN_ = 9.8 Hz). The signal of C6 did not show any ^13^C–^15^N splitting. Thus, analysis of the *J*_CN_ couplings allowed the straightforward determination of the structure of intermediate 100*.

**Scheme 34:**
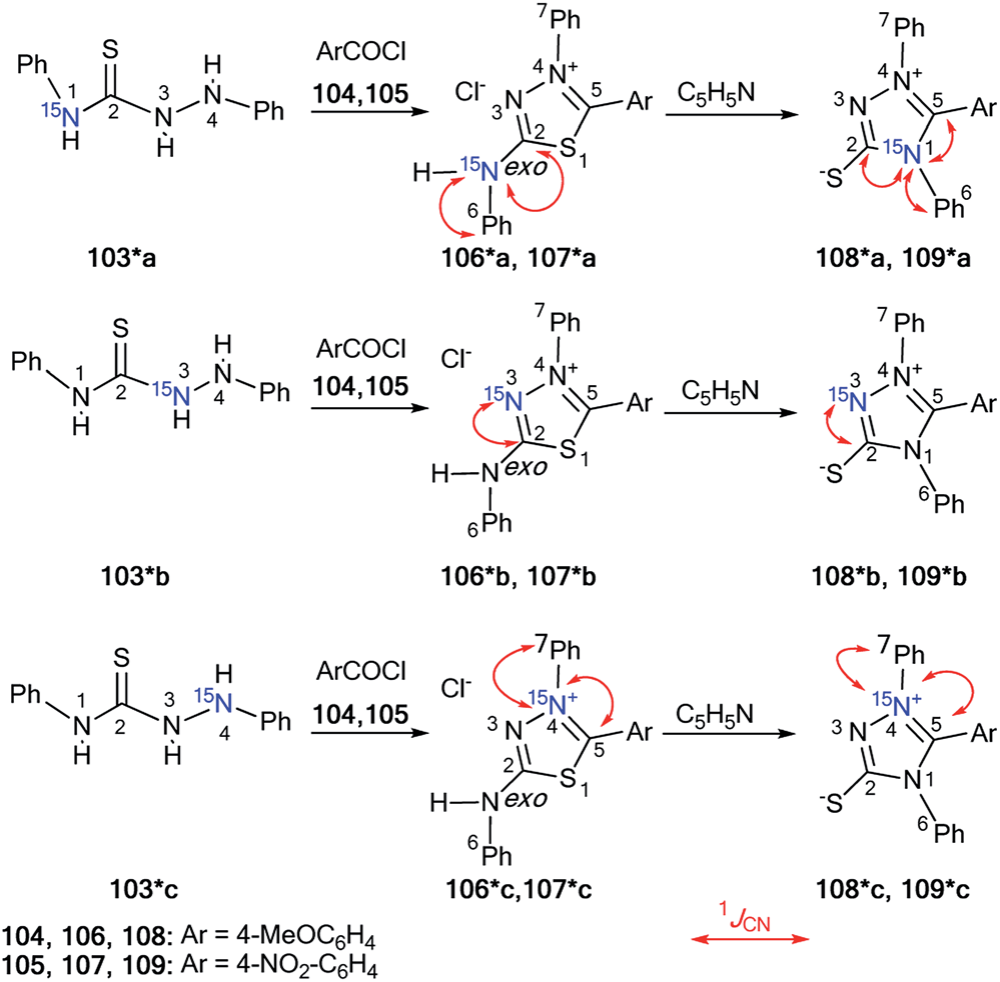
The use of ^15^N labels to establish the structure of triazoles and thiadizoles. The observed ^1^*J*_CN_ couplings are shown by red arrows. The measured ^1^*J*_CN_ values are presented in Table 4.

The measurement of direct^13^ C–^15^N SSCCs permitted the identification of the conversion of thiadiazoles 106*a–c and 107*a–c into 1,2,4-triazoles 108*a–c and 109*a–c, respectively (Scheme 34).^49^ This work is one example of the successful use of ^15^N-labeled samples for the investigation of the Dimroth-like rearrangement.

The three differently ^15^N-enriched 1,4-diphenylthiosemicarbazides 103*a–c in reaction with two different aroyl chlorides 104 and 105 were used as labeled starting materials. Thiosemicarbazide 103*a was synthesized by treatment of phenylhydrazine with ^15^N-phenyl isothiocyanate, which was the product of the interaction between ^15^N-aniline, carbon disulfide and ethyl chloroformate (equivalent quantities). To obtain compound 103*b/103*c, it was necessary to use labeled hydrazine obtained by the diazotization of aniline/^15^N-aniline with Na^15^NO_2_/NaNO_2_ in the reaction with phenyl isothiocyanate and perform the subsequent reduction in a mixture of Na_2_SO_3_–Na_2_S_2_O_3_. The formation of structures 106*a–c/107*a–c from compounds 108*a–c/109*a–c was observed by refluxing in pyridine solution. The measured values of ^1^*J*_CN_ for heterocycles 106*a–c- and 109*a–c were collected in Table 4.

**Table 4:** Observed direct ^13^C–^15^N coupling constants for compounds 106–109*a–c

Compound	^1^ *J*_CN_, Hz	
106*a	C2–N^*exo*^	23.1 ± 1.5
	C6–N^*exo*^	14.1 ± 1.5
106*b	C2–N3	<2
106*c	C5–N4	14.1 ± 1.5
	C7–N4	16.1 ± 1.5
107*a	C2-N^*exo*^	23.1 ± 1.5
	C6-N^*exo*^	14.1 ± 1.5
107*b	C2–N3	<1
107*c	C5–N4	16.1 ± 0.8
	C7–N4	16.1 ± 0.8
108*a	C2–N1	4.0 ± 1.5
	C5–N1	18.1 ± 1.5
	C6–N1	16.1 ± 1.5
108*b	C2–N3	3.0 ± 0.8
108*c	C5–N4	19.1 ± 0.8
	C7–N4	19.1 ± 0.8
109*a	C2–N1	4.0 ± 1.5
	C5–N1	20.1 ± 1.5
	C6–N1	16.1 ± 1.5
109*b	C2–N3	3.0 ± 0.8
109*c	C5–N4	20.1 ± 0.8
	C7–N4	18.1 ± 0.8

The main characteristics confirming the formation of structures 108* and 109* were the appearance of three direct ^13^C5–^15^N1 coupling constants that were observed in the carbon spectra of isotopomers 108*a and 109*a. The starting heterocycles 106*a and 107*a were only characterized by two ^13^C_2_–^15^N^*exo*^ and ^13^C6–^15^N^*exo*^ spin–spin interactions. The detection of the ^1^*J*_C5–N1_ couplings verified the transformation of compound 106*/107* into 108*/109* under reflux in pyridine.

### 3.2. Analysis of *J*_CN_ and *J*_HN_ couplings in the study of other ring-chain rearrangements and transformations occurring with changes to the heterocyclic scaffold

**Scheme 35:**
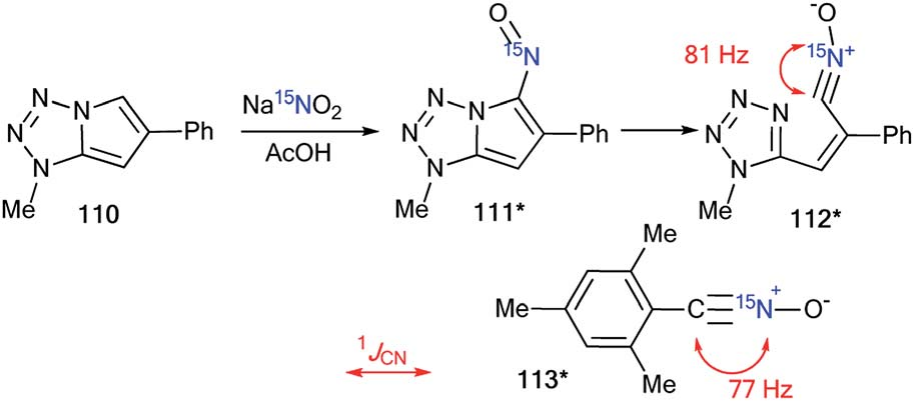
Determination of the structure of the nitrosation product using a ^15^N label. The observed ^1^*J*_CN_ couplings are shown by red arrows.

The analysis of the ^1^*J*_CN_ value was used for the determination of the structure of the nitrosation product obtained from compound 110 (Scheme 35).^50^ The data from the 1D ^1^H and ^13^C NMR spectra did not permit the identification of the ring opening of the pyrrole fragment in 110 under the action of sodium nitrite in acetic acid. The use of the ^15^N-labeled sodium nitrite and analysis of the direct ^13^C–^15^N coupling constant showed that the tetrazole derivative 111* underwent a transformation into the open form 112*. The conclusion was based on comparison of the ^13^C–^15^N spin–spin interaction (^1^*J*_CN_ = 81 Hz) that was observed in the carbon spectrum of compound 112* to that (^1^*J*_CN_ = 77 Hz) obtained for the earlier structure 113*.^51^

**Scheme 36:**
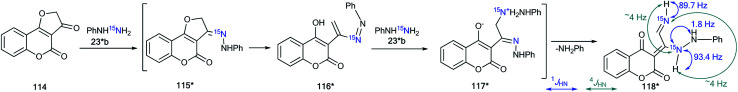
Determination of the positions of the ^15^N atoms in 118*. The observed direct and long-range ^1^H–^15^N coupling constants are shown by blue and green arrows, respectively.

The use of ^15^N-phenylhydrazine 23*b in the reaction of 2,3-dihydrofuro[3,2-*c*]coumarin-3-one 114 is another example of the use of labeled compounds to study chemical conversions in a series of heterocyclic compounds (Scheme 36).^52^ The analysis of the ^1^H–^15^N coupling constants allowed the determination of the positions of the labeled atoms in product 118* and proved the mechanism of the transformation. In the proton spectrum of compound 118*, direct and long-range ^1^H–^15^N spin–spin interactions were detected for two signals of ^15^NH-groups (^1^*J*_HN_ 89.7 Hz, ^4^*J*_HN_ ∼4 Hz and ^1^*J*_HN_ 93.4 Hz, ^4^*J*_HN_ ∼4 Hz). Moreover, additional splitting was observed for the protons from the CH=N and NH–Ph fragments. Thus, compound 118* was formed by the double addition of ^15^NH_2_–NH–Ph (23*b) and the elimination of aniline in the last step of the reaction. This mechanism involves the formation of intermediates 115*–117* (Scheme 36).

**Scheme 37:**
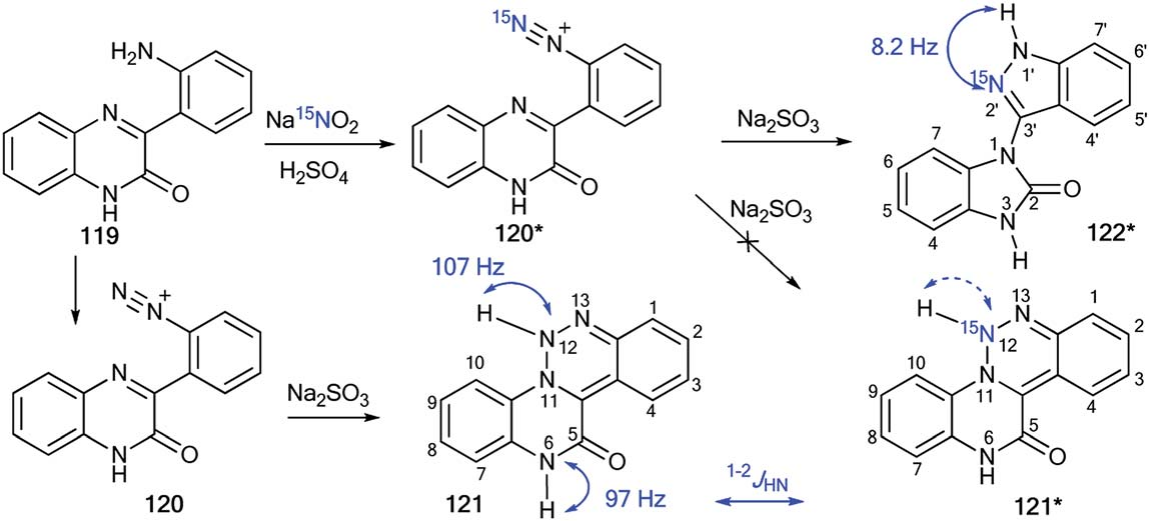
Establishment of the correct structure of 122* by using the ^15^N label. The observed ^1–2^*J*_HN_ couplings are shown by blue arrows. The expected but unobserved ^1^*J*_HN_ coupling is indicated by a dashed blue arrow.

The revision of the result for the reduction reaction of diazonium salt 120 that was obtained by the diazotation of amine 119 was described in another work.^53^ Previously, it was considered^54^ that the product of this transformation is 1,2,3-triazine 121 (Scheme 37), while the determination of structure 121 was based on the detection of two direct ^1^H–^15^N coupling constants (^1^*J*_H–N12_ = 107 Hz and ^1^*J*_H–N6_ = 97 Hz) in the 1D ^15^N NMR spectrum of the unlabeled sample. However, using ^15^N-labeled diazonium salt 120* in the reaction with sodium sulfite shed light on the real method of this transformation. The signal from the ^15^N-nitrogen atom in the resulting compound was only characterized by one ^1^H–^15^N coupling constant, ^2^*J*_HN_ = 8.2 Hz. Therefore, the 1,2,3-triazine structure of 121* was rejected, and it was shown that the reduction of compound 120* led to heterocycle 122*.

**Scheme 38:**
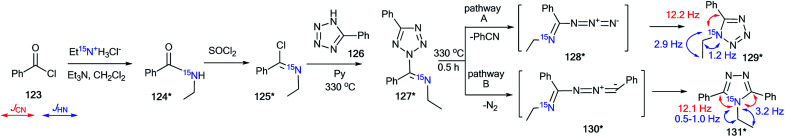
Analysis of ^13^C–^15^N and ^1^H–^15^N SSCCs in the NMR spectra of ^15^N-labeled products 129* and 131* to establish the reaction pathways. The observed ^13^C–^15^N and ^1^H–^15^N coupling constants are shown by red and blue arrows, respectively.

An unusual transformation was detected from the use of ^15^N-enriched compound 125* in a reaction with phenyltetrazole 126 (Scheme 38).^55^ The synthesis of chloride 125* included the interaction of benzoyl chloride and labeled ethylammonium chloride (^15^N, 99%) in the presence of triethylamine in dichloromethane. Then, amide 124* was treated with thionyl chloride. The interaction of 125* and 126 led to heterocycles 129* and 131*, which were characterized by ^13^C–^15^N and ^1^H–^15^N SSCCs.

These data confirmed the structures of compounds 129* and 131*. In the proton NMR spectra of 129* and 131*, the signals of the *N*-ethyl fragments showed additional splittings (^2^*J*_HN_ = 1.2 Hz, ^3^*J*_HN_ = 2.9 Hz and ^2^*J*_HN_ = 0.5–1.0 Hz, and ^3^*J*_HN_ = 3.2 Hz, respectively). Moreover, in the ^13^C NMR spectra, the signals of the carbons of the tetrazole and 1,2,4-triazine fragments were detected as doublets with magnitudes of 12.2 Hz and 12.1 Hz, respectively. These results indicate that the formation of structures 129* and 131* occurred over two different mechanisms (pathways A and B, Scheme 38). However, these transformations started with the common intermediate 127*. Pathway A included the elimination of benzonitrile, obtaining azide 128*, which underwent cyclization into tetrazole 129*. Compound 131* may be formed according to the mechanism demonstrated by route B. In this case, structure 127* transformed into 130* by the elimination of nitrogen. Then, the isomerization of intermediate 130* led to 1,2,4-triazine 131*.

**Scheme 39:**
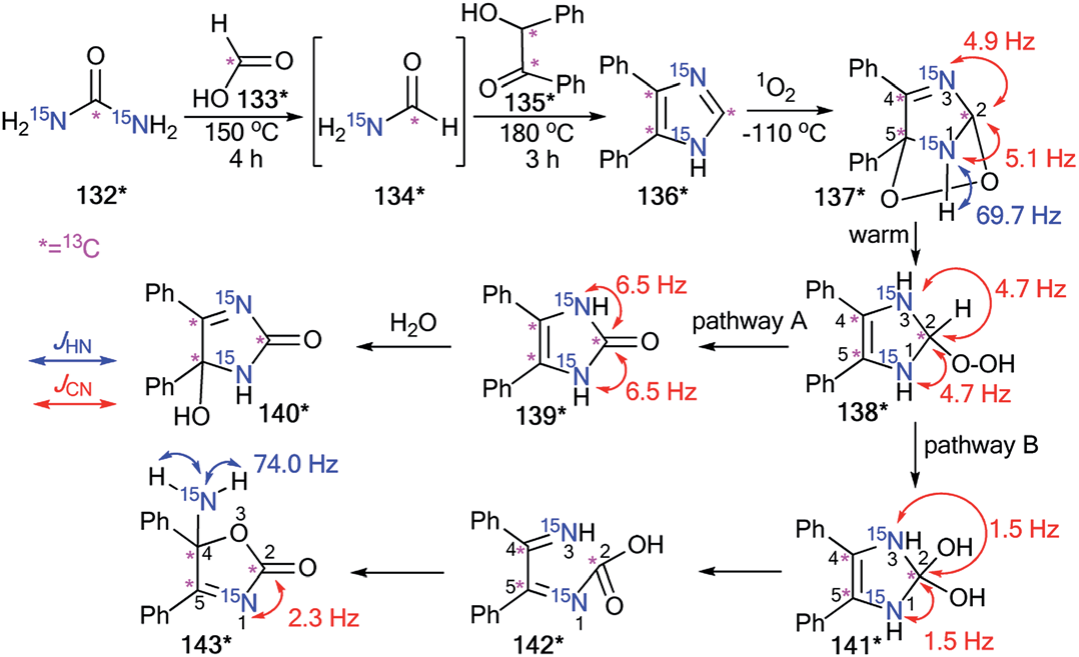
The analysis of the ^13^C- and ^15^N-labeled intermediates for the choice between possible reaction pathways. The observed direct ^13^C–^15^N and ^1^H–^15^N coupling constants are shown by red and blue arrows, respectively.

The incorporation of the ^13^C and ^15^N atoms in compound 136* allowed determination of the method of the photosensitized oxidation of imidazole derivatives (Scheme 39).^56^ This interaction with singlet oxygen was considered a model reaction for natural and biologically active structures containing the imidazole fragment (guanosine, xanthine, theophylline, histidine, *etc.*). The production of 136* involved heating a mixture of ^15^N_2_-urea 132* (^13^C, 99% and ^15^N, 98%) and ^13^C-formic acid 133* (^13^C, 99%) at 150 °C for 4 h (Scheme 39). Then, ^13^C-enriched benzoin 135* was introduced, and the reaction mixture was heated at 180 °C.

The synthesis of labeled benzoin 135* was based on coupling 2 mol of ^13^C-benzaldehyde (^13^C, 99%) in the presence of NaCN. The photosensitized oxidation of ^13^C_3_,^15^N_2_-labeled 4,5-diphenylimidazole 136* was carried out in an NMR tube. This experiment permitted the observation of the formation of several intermediates 137*–141* that were obtained from 136* under the action of singlet oxygen.

Oxidation of labeled imidazole 136* gave one intermediate 137* at −100 °C. The observation of splitting of C2 (^1^*J*_C–N1_ = 5.1 Hz and ^1^*J*_C–N3_ = 4.9 Hz) showed that this carbon bonds with two nonequivalent, labeled nitrogen atoms. Moreover, a ^1^H–^15^N1 SSCC of 69.7 Hz was detected in the proton spectrum for this intermediate. These data and an analysis of the chemical shifts of the labeled atoms with the measurement of *J*_CN_ couplings confirmed the formation of 2,5-endoperoxide 137* in the reaction of 136* with singlet oxygen. The warming of 136* led to decomposition, which could occur in two pathways: A and B (Scheme 39).

Both paths contained general intermediate 138*, which was obtained by the transformation of 137*. In the first pathway, 138* lost water to give 139*, which underwent hydrolysis to form 140*. Another pathway included the transformation of 138* into diol 141*. Then, intermediate 141* rearranged to 143* by the opening and reclosing of the imidazole ring. This transformation occurred through the open form 142*. The formation of intermediates 138*–140* and 141*, 143* was monitored by ^1^H, ^13^C and ^15^N NMR spectroscopy.

It should be noted that the estimation of the ^13^C–^15^N and ^1^H–^15^N coupling constants with analysis of the chemical shifts for ^1^H, ^13^C and ^15^N NMR spectra and the measurement of the ^1^H–^13^C spin–spin interaction allowed for the determination of the structures of these compounds. For example, the detection of only one ^1^*J*_CN_ = 2.3 Hz for C2 in the carbon spectra of 143* showed that one bond of C2–^15^N was broken. The chemical shift of C4 was characteristic of a sp^3^-carbon connected to two heteroatoms. These data indicated that C4 was bonded to an oxygen and the N1 nitrogen. Moreover, the signal from the amino group was observed as a triplet split by two bonded hydrogens (^1^*J*_H–N1_ = 74.0 Hz) in the proton spectrum. This information suggested that the five-membered imidazole ring in 141* opened and reclosed to form intermediate 143*.

**Scheme 40:**
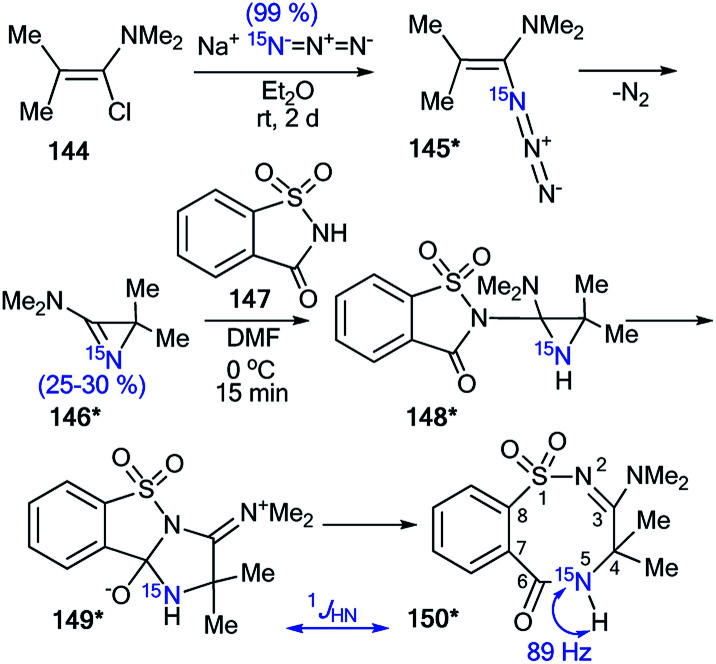
Determination of the ^15^N atom positions in 150*. The observed direct ^1^H–^15^N coupling constant is shown by the blue arrow.

**Scheme 41:**
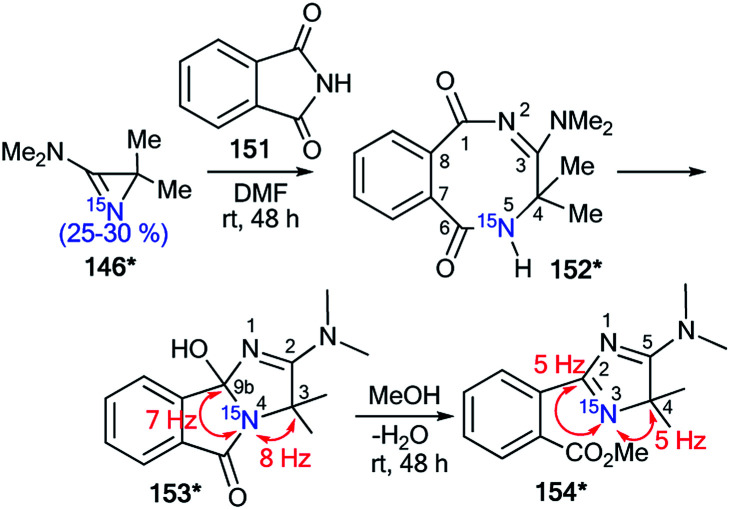
Establishment of the reaction mechanism for the reaction of azirine 146* with phthalimide 151. The observed ^13^C–^15^N coupling constants are shown by red arrows.

The application of ^15^N-azirine 146* (^15^N, 50%) with one equivalent of unlabeled 2,2-dimethyl-3-(dimethylamino)-2*H*-azirine 146 provided an opportunity to illustrate the capacity of ^15^N-labeling and ^1^H–^15^N and ^13^C–^15^N analysis in studying the mechanisms of chemical transformation in a series of three-membered heterocyclic compounds (Schemes 40 and 41).^57^ The synthesis of compound 146* was based on the treatment of trimethyl-1-propenylamine 144 with enriched sodium azide (^15^N, 99%). The interaction of 146* (^15^N, 25–30%) with 1,1-dioxo-1,2-benzothiazol-3-one (saccharine) 147 led to compound 150*, which was characterized by ^1^*J*_HN_ = 89 Hz (Scheme 40). This result confirmed the position of the ^15^N-labeled atom in 150* and showed that the transformation of 144* into 150* occurs *via* aziridine 148* and ring expansion to zwitterion 149* (Scheme 40).

In the case of the reaction between azirine derivative 146* and phthalimide, the tricyclic product 153*^57^ was isolated. The structure of 153* was identified by measurements of ^13^C–^15^N SSCCs (^1^*J*_C9b–N2_ = 7 Hz and ^1^*J*_C3–N2_ = 8 Hz), which were registered for sp^3^-hybridized carbon atoms (Scheme 41). Additionally, the ^1^*J*_HN_-couplings were not detected in the 1D ^1^H and ^15^N NMR spectra. The authors suggested that compound 153* is the product of the transformation of 2,3-dihydro-2,5-benzodiazocine-1,6-dione 152*. Moreover, the analysis of chemical shifts for the carbon atoms and ^13^C–^15^N coupling constants of signals C2 (^1^*J*_CN_ = 5 Hz) and C4 (^1^*J*_CN_ = 5 Hz) verified the formation 2-(4*H*-imidazol-2-yl)benzoic acid derivative 154*, which was obtained by treatment of 153* with methanol.

**Scheme 42:**
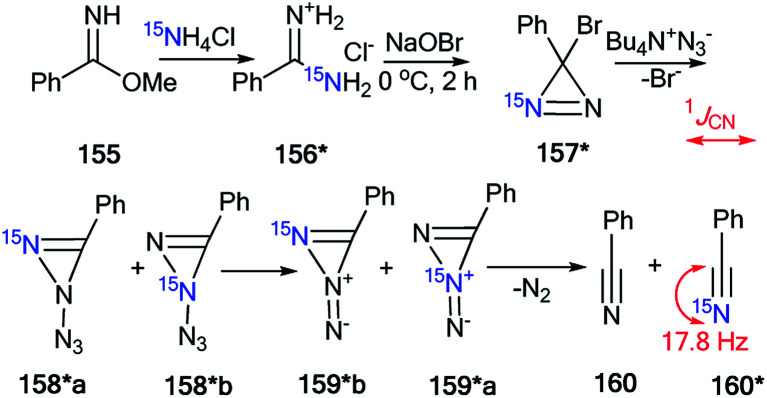
The use of ^15^N-labeled diazirine 157* for the determination of the reaction pathway. The observed ^13^C–^15^N coupling constant is shown by the red arrow.

An interesting example of the use of ^15^N-labeled compounds for determining the ring open reaction pathway in a series of diazirines was reported by Creary and co-workers.^58^ The incorporation of ^15^N in structure 157* was based on the reaction of methyl benzimidate 155 with enriched ammonium chloride (^15^N, 99%) (Scheme 42). Then, oxidation of the resulting benzamidine 156* with NaOBr yielded ^15^N-phenylbromodiazirine 157*. Compound 157* reacted with tetrabutylammonium azide to give a mixture of benzonitriles 160 and 160* in a ratio of 1 : 1. The ^13^C NMR spectrum confirmed partial ^15^N labeling for the obtained product. The nitrile carbon of 160* appeared as a doublet (^1^*J*_CN_ = 17.8 Hz), while the carbon of the cyano group of 160 registered as a singlet. A comparison of the intensity of these signals showed a 50% excess of the ^15^N isotope of 160*. This result proved that the formation compounds 160 and 160* from diazirine 157* and BuN^+^N_3_^−^ occurred *via* the structures 158*a,b and 159*a,b.

Thus, analysis of the literature shows that the measurement of the ^1^H–^15^N and ^13^C–^15^N coupling constants can be successful for the determination of different mechanisms of chemical transformations and rearrangements in nitrogen-containing heterocycles.

## 4. ^13^C–^15^N and ^1^H–^15^N coupling constants as an approach to the determination of structural reaction/isomerization products occurring without changes to the heterocyclic core

Nitrogen-enriched heterocycles are molecules containing several reaction centers. This class of compounds can exhibit dual behaviors, expressed by the fact that they can react with both nucleophilic and electrophilic reagents. As a result, such interactions often give a mixture of isomers even when the heterocyclic scaffold does not undergo rearrangement. This situation requires that researchers use different methods for the determination of structures obtained from similar products. One of the effective approaches that can be used to solve these problems is the measurement of the *J*_CN_ and *J*_HN_ couplings in ^15^N-labeled samples. In this part of the review, examples of ^15^N labeling and the use of the analysis of ^1^H–^15^N and ^13^C–^15^N coupling constants for the determination of functionalization methods that do not lead to changes in the heterocyclic core were collected. Such chemical transformations can be observed in reactions of *N*-alkylation, nitration, amination and obtaining azomethines based on different heterocycles.

### 4.1. Using *J*_CN_ and *J*_HN_ couplings for the investigation of *N*-alkylation reactions

**Scheme 43:**
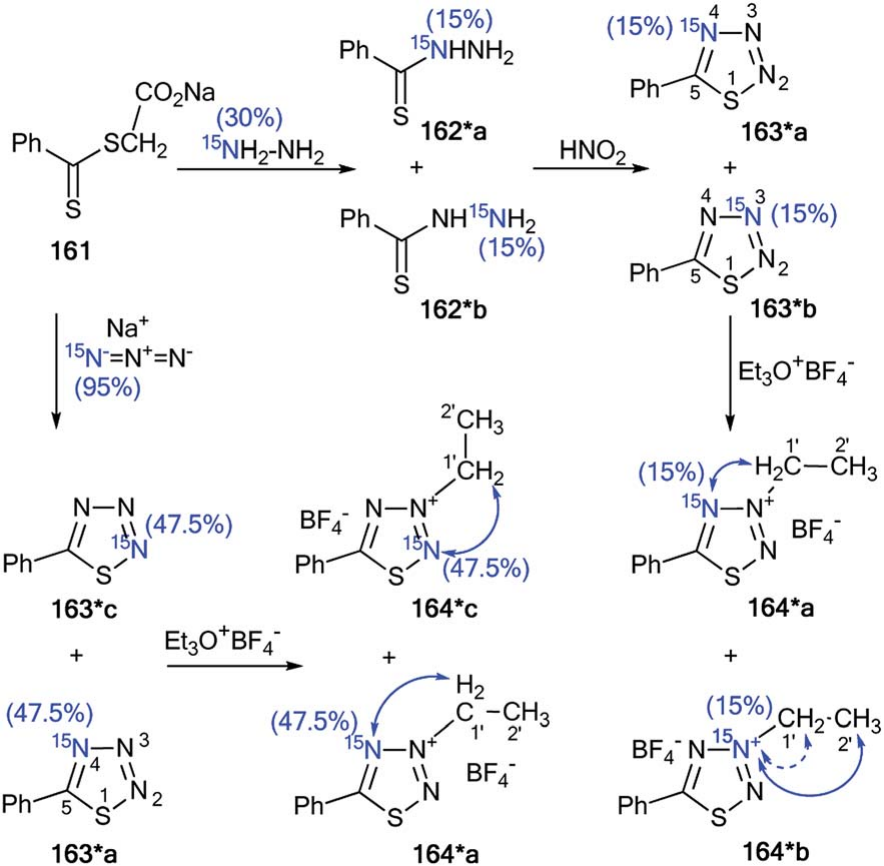
The use of ^15^N-labeled thiatriazoles to determine the site of alkylation. The observed ^3^*J*_HN_ couplings are shown by blue arrows. The expected but unobserved ^2^*J*_HN_ coupling is indicated by a dashed blue arrow. The values of the observed vicinal ^1^H–^15^N coupling constants are shown in Table 5.

The incorporation of ^15^N atoms in different positions of 5-phenyl-1,2,3,4-thiatriazole 163 allowed for determination of the structure of the products obtained by alkylation with triethyloxonium tetrafluoroborate (Scheme 43).^59^ The synthesis of isotopomers 163*a,b was based on the reaction of a sodium salt of carboxymethyl dithiobenzoate 161 with ^15^N-hydrazine (^15^N, enrichment 30%) and the subsequent interaction with nitric acid. A mixture of 15% isotopically labeled compounds 163*a,b was obtained.

**Fig. 2:**
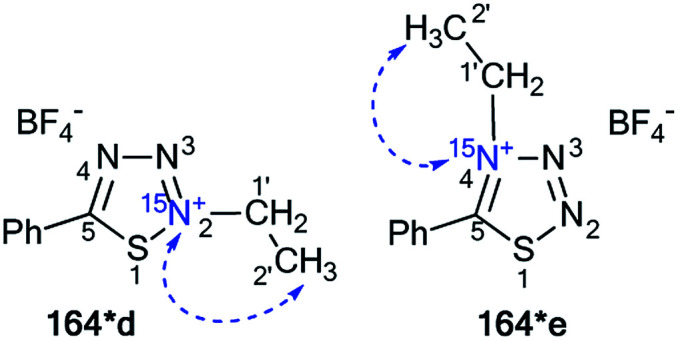
The two possible alternative *N*-alkylation products are illustrated by structures 164*d and 164*e (*N*-2 and *N*-4-alkylation, respectively). The expected but unobserved ^3^*J*_HN_ couplings are indicated by dashed blue arrows.

Treatment of salt 161 with ^15^N-labeled sodium azide (95%, ^15^N) led to the formation of a mixture of compounds 163*a (^15^N, 47.5%) and 163*c (^15^N, 47.5%). The use of the mixture of 163*a and 163*b/163*a and 163*c in a reaction with EtO_3_^+^BF_4_ gave *N*-alkylated derivatives 164*a and 164*b/164*a and 164*c (Scheme 43). The position of the alkylation of 1,2,3,4-thiatriazole with ethyl fragments in 164*a–c was determined by analysis of the ^1^H–^15^N coupling constants. It should be noted that vicinal (^3^*J*_HN_) couplings were only registered in the proton spectra of 164*a–c, and the expected geminal coupling (^2^*J*_HN_) was not detected by 1D ^1^H NMR spectroscopy due its small amplitude (approximately 1.2 Hz). For compound 164*a/164*c, ^3^*J*_H1′–N2_/^3^*J*_H1′–N2_ was measured (Table 5). The detection of an additional splitting ^3^*J*_H2′–N3_ (3.7 Hz) for the methyl signal of 164*b unambiguously confirmed the structures of the products obtained by the interaction of compound 164*a–c with EtO_3_^+^BF_4_ because the formation of the alternative isomers 164*d,e should lead to the appearance of ^3^*J*_H2′–N2_ and ^3^*J*_H2′–N4_ couplings, respectively (Fig. 2).

**Table 5:** Vicinal ^1^H–^15^N coupling constants measured in the 1D ^1^H NMR spectra of 147*a–c

Compound	^3^ *J*_HN_, Hz
164*a	1.64 ± 0.05
164*b	3.74 ± 0.1
164*c	2.3 ± 0.05

**Scheme 44:**
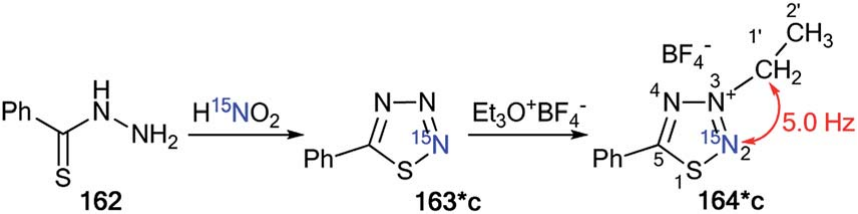
The use of ^15^N-labeled thiatriazoles to determine the site of alkylation. The observed ^2^*J*_C1′–N2_ coupling is shown by a red arrow.

Moreover, the authors developed a selective synthesis for isotopomer 164*c. This approach included the interaction of unlabeled 2-ethylthiobenzoylhydrazine 162 with ^15^N-nitric acid. Then, the obtained 163*c underwent alkylation with triethyloxonium tetrafluoroborate (Scheme 44). The detection of ^2^*J*_C1′–N2_ = 5.0 Hz was further evidence for the binding of the *N*-ethyl fragment with the N2 atom in compounds 164*a–c.

**Scheme 45:**
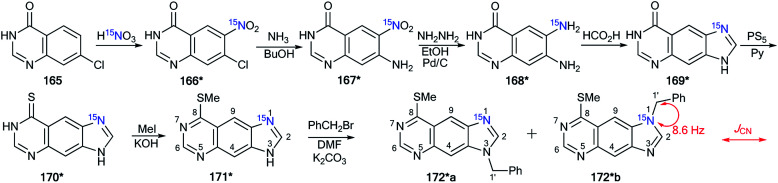
The use of ^15^N-labeled annulated imidazole to determine the site of alkylation. The observed ^1^*J*_C1′–N1_ coupling is shown by a red arrow.

Another example of the application of ^13^C–^15^N SSCC has been presented for the determination of the structures of *N*-alkylated derivatives 172*a and 172*b that were obtained by the interaction of ^15^N-labeled 8-methylthioimidazo[4,5-*g*]quinazoline 171* with benzyl bromide.^60^ The synthesis of compound 171* included the treatment of 7-chloro-4-quinazolone 165 with ^15^N-nitric acid (^15^N, 99%) (Scheme 45) to obtain ^15^N-labeled product 166*. The remainder of the synthesis followed Scheme 45 through heterocycles 167*, 168*, 169* and 170*. The chosen method for the synthesis of 171* determined the position of the ^15^N-labeled atom.

The use of 171* in the *N*-benzylation reaction led to the formation of two isomers, 172*a and 172*b, that were separated by column chromatography. The determination of the site of the *N*-alkyl fragments in compounds 172*a and 172*b was based on data from the proton-decoupled ^13^C NMR spectra. In the case of isomer 172*a, a signal of atom C1′ of a benzyl moiety was registered as a singlet. The benzylic carbon of 172*b showed splitting and appeared as a doublet (^1^*J*_CN_ = 8.6 Hz) due to the spin–spin interaction ^13^C1′–^15^N1 (Scheme 45).

The obtained results allowed the authors to reach the conclusion that this method for determining the site of alkylation may well prove applicable in other heterocyclic systems where ^15^N can be selectively incorporated.

**Scheme 46:**
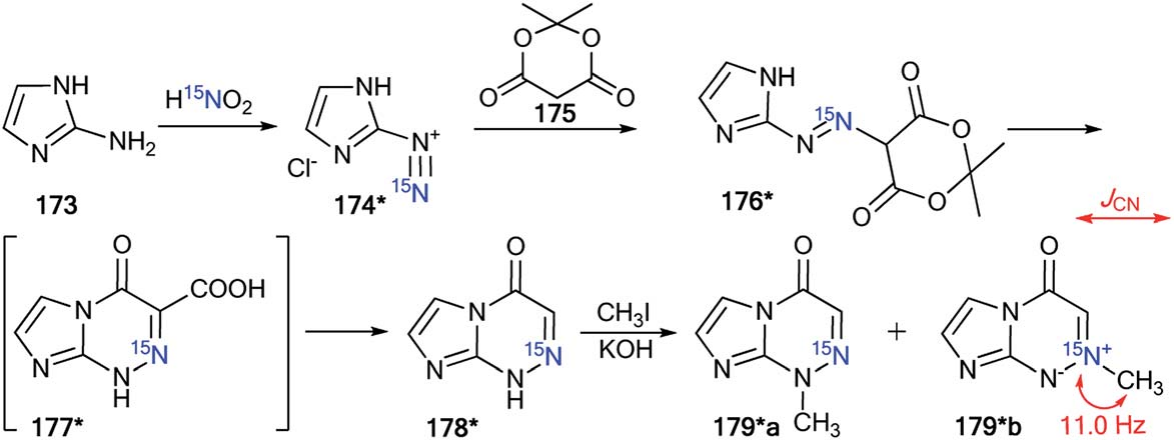
The use of ^15^N-labeled triazine to determine the site of alkylation. The observed ^1^*J*_CN_ coupling is shown by a red arrow.

Indeed, the selective ^15^N-labeling of compound 178* and analysis of the ^13^C–^15^N spin–spin interaction allowed the unambiguous determination of *N*-methylation sites in a series of imidazo[2,1-*c*][1,2,4]triazin-4-ones and related azolo[5,1-*c*][1,2,4]triazin-4-ones.^61^ In this work, compound 178* was prepared by the coupling of isopropylidene malonate 175 (Meldrum's acid) with diazoazole 174* (Scheme 46). The incorporation of the isotope label in structure 174* was based on the treatment of 2-aminoimidazole 173 with ^15^N-enriched sodium nitrite (^15^N, 36%) in an acidic medium. The reaction between 175 and 174* gave product 176*. The heating of 176* in acetic acid led to azoloazine 178*. Cyclization and decarboxylation occurred in one pot, as shown in Scheme 46.

The methylation of compound 178* yielded isomers 179*a and 179*b, which were separated. The detection of the direct ^13^C–^15^N coupling of 11.0 Hz for the signal of the carbon of the *N*-methyl group confirmed the formation of betaine-like structure 179*b in the alkylation reaction of imidazo[2,1-*c*][1,2,4]triazin-4-ones and their analogs.

It should be noted that today, for the determination of the site(s) of the benzylation/methylation of an unlabeled analog 171*/178*, it is possible to use conventional ^1^H, ^13^C-NMR methods because for the resulting products, the ^1^H–^13^C spin–spin interactions between the protons of the N–CH_2_/N–CH_3_ fragment and the carbons of the heterocyclic part (8-methylthioimidazo[4,5-*g*]quinazoline/imidazo[2,1-*c*][1,2,4]triazin-4-one) can be identified. That is, these cases do not require ^15^N labeling and the analysis of *J*_CN_ couplings. However, the determination of *N*-adamantylation site(s) in unenriched analogs of heterocycle 178* such as azolo[5,1-*c*][1,2,4]triazine, azolo[1,5-*a*]pyrimidine and related azoloazines using well-established ^1^H and ^13^C NMR methods (such as 1D, 2D COSY, HMQC, HMBC, and INADEQUATE spectra) are difficult because the heterocyclic moiety is covalently attached to the adamantane tertiary carbon that has no bond with hydrogen atoms. Nuclear Overhauser effect spectroscopy (NOESY or ROESY) also does not provide unequivocal structures for *N*-adamantylated derivatives. In this case, the application of ^15^N labeling and the measurement of ^13^C–^15^N and ^1^H–^15^N coupling constants allows for the determination of the *N*-adamantylation sites in the azolo-1,2,4-triazine and 1,2,4-triazolo[1,5-*a*]pyrimidine series.

**Scheme 47:**
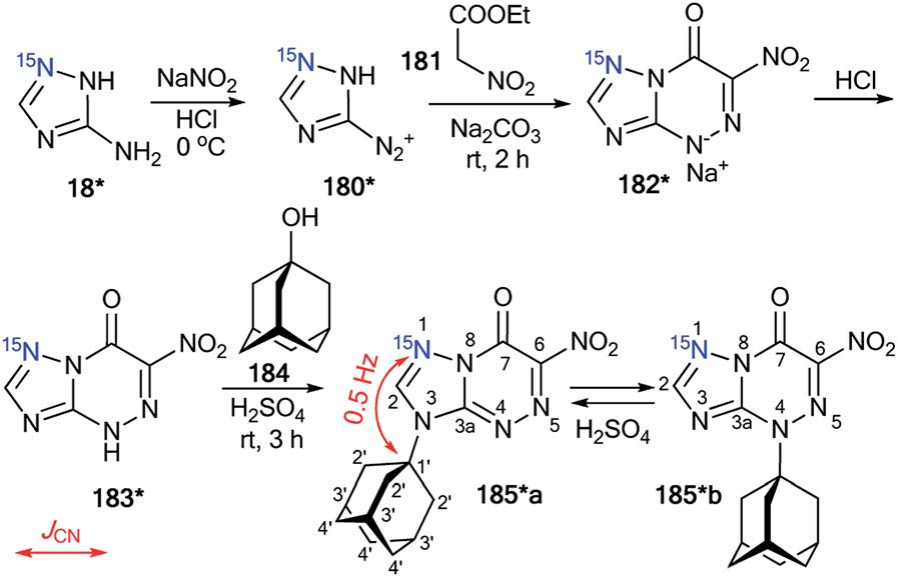
The use of ^15^N-labeled triazolotriazine to determine the site of adamantylation. The observed ^3^*J*_CN_ coupling is shown by a red arrow.

The first time efficiency of this approach was shown in the study of the interaction of 6-nitro-1,2,4-triazolo[5,1-*c*][1,2,4]triazin-7-one 183* (Scheme 47).^62^ The synthesis of 183* was based on the treatment of amine-1,2,4-triazole 18* (^15^N, 87%) with sodium nitrite in acidic medium and a subsequent interaction of compound 180* with ethyl nitroacetate 181.

Then, the resulting sodium salt 182* was transformed into azoloazine 183*, which was treated with adamantanol 184 in a solution of sulfuric acid. The adamantylation of 183* gave a mixture of isomers 185*a and 185*b. The appearance of the ^15^N label in structure 185*a permitted the determination of the conformation of the additional adamantane fragment in the N3-atom azole rings by the observation of the ^3^*J*_CN_ couplings (0.5 Hz) in the carbon spectrum.

The structure of the adamantylated derivative 185*b was determined by ^13^C NMR spectroscopy *via* comparison with a model compound, *N*-methylated 1,2,4-triazolo[5,1-*c*][1,2,4]triazin-7-one. Moreover, the use of ^15^N-enriched azoloazine 183* in the investigation of adamantylation showed that compound 185*b is a product of the reversible isomerization of 185*a; this rearrangement occurs *via* the formation of an adamantyl cation and heterocyclic base 183*.

**Scheme 48:**
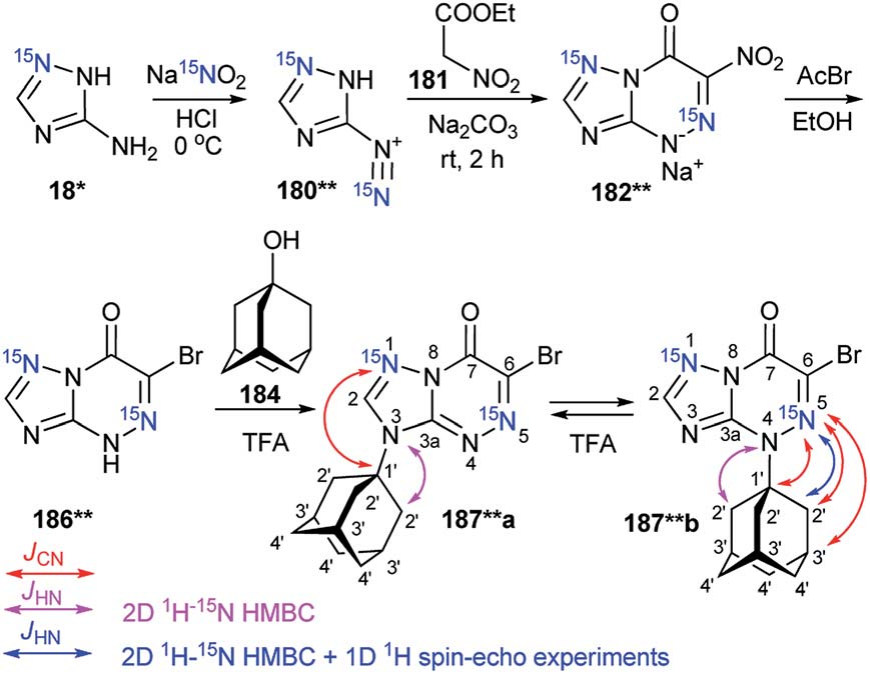
The use of double ^15^N-labeled triazolotriazine to determine the site of adamantylation. The measured *J*_CN_ couplings are shown by red arrows. The *J*_HN_ couplings measured by amplitude-modulated 1D ^1^H spin-echo experiments and detected in the 2D ^15^N-HMBC spectra are shown by blue and magenta arrows (see the legend in the scheme).

The selective incorporation of two ^15^N atoms in different positions of the 1,2,4-triazolo[5,1-*c*][1,2,4]triazin-7-ones and other nitrogen-containing heterocycles together with a combined analysis of the *J*_HN_ and *J*_CN_ coupling constants was more effective for the structural determination of heterocyclic *N*-adamantylated derivatives.^39^ For example, the use of this approach allowed for the determination of the structures of compounds 187**a and 187*b obtained by the coupling between 186** and 184 in trifluoroacetic acid (TFA) solution under reflux (Scheme 48).

The incorporation of two labeled atoms in compound 186** was based on the treatment of ^15^N-3-amino-1,2,4-triazole 18* (^15^N, 98%) with labeled sodium nitrite (^15^N, 98%). The reaction of 18* and ^15^N-sodium nitrite in acidic medium allowed the *in situ* production of diazonium salt 180**, which reacted with ethyl nitroacetate 181 in a sodium carbonate solution. This reaction led to the formation of ^15^N_2_-1,2,4-triazolo[5,1-*c*][1,2,4]triazinone 182**. The interaction of 182** with hydrogen bromide generated from acetyl bromide and ethanol gave compound 186**.

It should be noted that the formation of an isomeric mixture in the adamantylation reaction of 186** could explain the rearrangement of the N3-isomer 170**a into the N4-isomer 170**b. This process could include the production of an adamantyl cation and the base of 186**. The structures of compounds 187**a,b were unambiguously confirmed by analysis of the *J*_CN_ and *J*_HN_ couplings. The detection of a single ^3^*J*_C1′–N1_ coupling (0.4 Hz) for the adamantane carbon in compound 187**a indicated that the substituent group is attached to the N3 atom of the 1,2,4-triazole ring (Table 6 and Scheme 48).

**Table 6:** *J*_CN_ couplings (Hz) observed from the signals of the adamantyl fragment in the spectra of azoloazines 187**a,b, 191** and 192**a,b^a^

Compound	^*n*^ *J*_CN_, Hz
187**a	^3^ *J*_C1′–N1_ 0.4
187**b	^2^ *J*_C1′–N5_ 5.0
	^3^ *J*_C2′–N5_ 1.7
	^4^ *J*_C3′–N5_ 0.4
191**	^3^ *J*_C1′–N1_ 0.4
	^3^ *J*_C1′–N8_ 0.6
192**a	^1^ *J*_C1′–N2_ 6.5
	^2^ *J*_C1′–N3_ 3.8
	^2^ *J*_C2′–N2_ 0.4
	^3^ *J*_C2′–N3_ 1.2
	^3^ *J*_C3′–N2_ 1.6
	^4^ *J*_C3′–N3_ 0.2
	^4^ *J*_C4′–N2_ 0.3
192**b	^2^ *J*_C1′–N2_ 2.7
	^3^ *J*_C1′–N3_ 0.3
	^3^ *J*_C2′–N2_ 1.1
	^4^ *J*_C3′–N2_ 0.3

a The ^13^C–^15^N *J* coupling constants were measured by line-shape analysis in the 1D ^13^C spectra and acquired with selective ^15^N decoupling and broadband ^1^H decoupling. The estimated error in the *J*_CN_ values is 0.1 Hz, and the lower limit of reliable *J*_CN_ measurements is 0.2 Hz.

The attachment of the adamantane fragment to the N4 atom of the triazine ring in compound 187**b led to a large set of observable *J*_CN_ couplings, including geminal (^2^*J*_C1′–N5_ = 5.0 Hz) and vicinal (^3^*J*_C2′–N5_ = 1.7 Hz) (Table 6 and Scheme 45). The ^15^N-HMBC spectra of compounds 187**a,b allowed for ^3^*J*_HN_ couplings of the nitrogen atoms at a natural isotopic abundance. The analysis of the ^1^H–^15^N_3_/^15^N_5_ spin–spin interactions also confirmed the structures of the products of adamantylation of 186** (Table 7 and Scheme 48). Moreover, the appearance of the ^15^N isotope in the azine ring of 187**b permitted the observation of the long-range ^1^H2′–^15^N5 coupling constant (Table 7 and Scheme 48). This characteristic was additional evidence for the attachment of the adamantane substituent to the unlabeled N4 atom.

**Table 7:** *J*_HN_ couplings (Hz) of adamantylated azoloazines 187**a,b, 191** and 192**a,b^a^^,^^b^

Compound	N1/N2	N3/N5/N8	^15^N at natural abundance N1/N3/N4
187**a			^3^ *J*_H2′–N3_ (m)
187**b		^4^ *J*_H2′–N5_ 0.06 (w)	^3^ *J*_H2′–N4_ (m)
191**			^3^ *J*_H2′–N3_ (m)
192**a	^3^ *J*_H2′–N2_ 0.83 (s), ^4^*J*_H3′–N2_ 0.60 (m), ^5^*J*_H4′–N2_ 0.23 (m)	^4^ *J*_H2′–N3_ 0.06 (w), ^5^*J*_H3′–N3_ 0.11 (−)	
192**b	^4^ *J*_H2′–N2_ < 0.04^c^ (−), ^5^*J*_H3′–N2_ 0.04 (−)	^5^ *J*_H2′–N3_ < 0.04^c^ (w)	^3^ *J*_H2′–N1_ (m)

a The *J*_HN_ values were measured using amplitude-modulated 1D ^1^H spin-echo experiments with delays for the evolution of *J*_HN_ up to 1 s. The estimated error in the *J*_HN_ values is 0.02 Hz, and the lower limit of reliable *J*_HN_ measurements is 0.04 Hz.

b The cross-peaks in the 2D ^15^N-HMBC spectra were classified into three categories (weak – w; medium – m; strong – s). Weak peaks approximately correspond to *J*_HN_ < 0.5 Hz, strong peaks approximately correspond to *J*_HN_ > 2 Hz, and medium peaks correspond to the remaining values.

c The measurement of the *J*_HN_ values was impossible due to the fast transverse relaxation of the corresponding ^1^H nuclei.

**Scheme 49:**
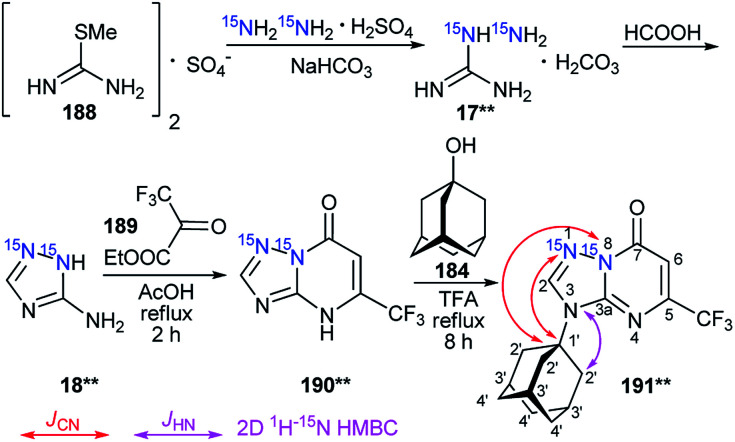
The use of double ^15^N-labeled triazolopyrimidine to determine the site of adamantylation. The measured *J*_CN_ couplings are shown by red arrows. The observed *J*_HN_ coupling in the 2D ^15^N-HMBC spectra is shown by a magenta arrow (see the legend in the scheme).

**Scheme 50:**
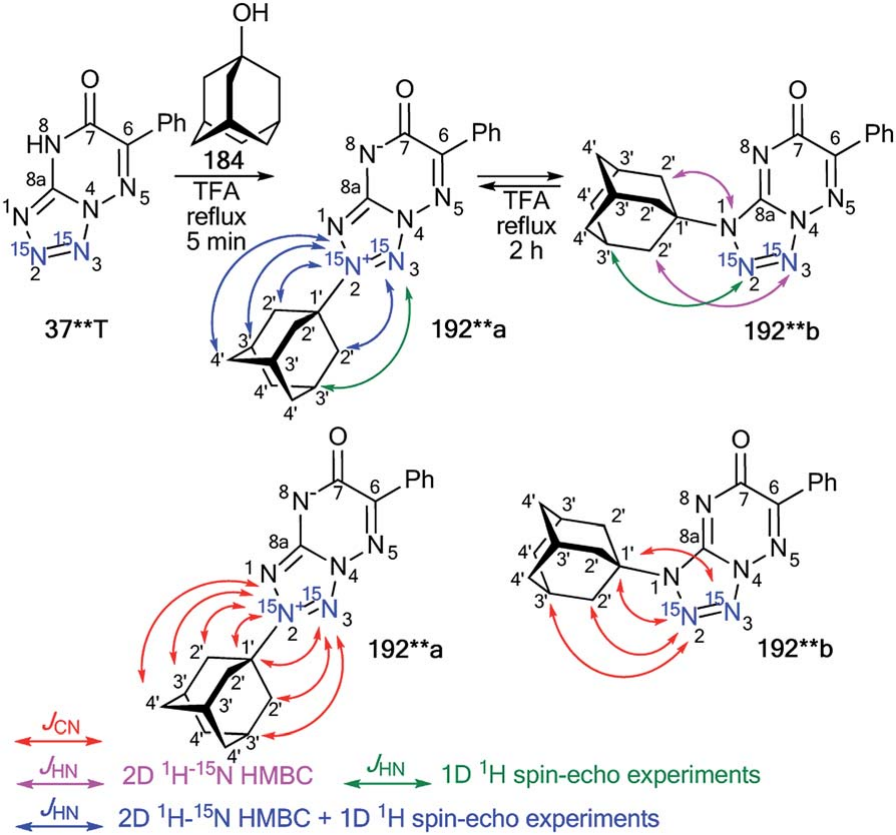
The use of double ^15^N-labeled triazolotetrazine to determine the site of adamantylation. The observed *J*_CN_ couplings are shown by red arrows. The measured *J*_HN_ couplings by amplitude-modulated 1D ^1^H spin-echo experiments and detected ^1^H–^15^N spin–spin interactions in the 2D ^15^N-HMBC spectra are shown by blue, green and magenta arrows (see the legend in the scheme).

In the abovementioned work,^39^ the selective incorporation of two ^15^N atoms and combined analysis of *J*_CN_ and *J*_HN_ were also used for the determination of the adamantylation site of 1,2,4-triazolo[1,5-*a*]pyrimidine and tetrazolo[1,5-*b*][1,2,4]triazine derivatives (Schemes 49 and 50).

In the first case, double-labeled amino-1,2,4-triazole 18** was used for to incorporate ^15^N in the core of 1,2,4-triazolo[1,5-*a*]pyrimidine. Compound 18** was synthesized by the interaction of ^15^N_2_-hydrazine sulfate (98%, ^15^N) with *S*-methyl isothiourea sulfate 188 and the consecutive cyclization of ^15^N_2_-aminoguanidine 17** with formic acid (Scheme 49). The use of 18** in the reaction with ethyl 4,4,4-trifluoroacetoacetate 189 yielded azoloazine 190**, containing two isotopic labels. The adamantylation of 190** in TFA solution was regioselective and led to the formation of only the N3-isomer 191**. The observed ^13^C1′–^15^N1/N8 coupling in the 1D ^13^C NMR spectra were acquired with selective decoupling from ^15^N1/^15^N8 and the detected ^1^H2′–^15^N3 cross-peak in the ^15^N-HMBC spectrum that unambiguously confirmed the position of the adamantyl fragment in compound 191* (Tables 6, 7 and Scheme 49).

In contrast to 190**, the adamantylation of tetrazolo-1,2,4-triazine 37**T in TFA solution led to N2- and N1-regioisomers (compounds 192**a and 192**b, respectively, Scheme 50). It was found that compound 192**b is the product of the reversible isomerization of 192*a. This process occurs *via* the formation of an adamantyl cation and heterocyclic base 37**T.

Compound 192**a was characterized by a set of ^1^H–^15^N coupling constants that were detected for the H2′ (^3/4^*J*_H2′–N2/N3_ = 0.83/0.06 Hz), H3′ (^4/5^*J*_H3′–N2/N3_ = 0.60/0.11 Hz) and H4′ (^5^*J*_H4′–N2_ = 0.23 Hz) atoms of the adamantane fragment (Table 7 and Scheme 50). These spin–spin interactions, with the exception of ^5^*J*_H3′–N3_, were also observed in the 2D ^15^N-HMBC spectrum. However, analysis of the *J*_HN_ couplings did not allow the determination of the adamantylation site. This problem was solved by measuring the ^13^C–^15^N coupling constant (Table 6 and Scheme 50). Observation of the direct ^1^*J*_C1′–N2_ (6.5 Hz) and other ^13^C–^15^N interactions for the C1′ (^2^*J*_C–N3_ = 3.8 Hz), C2′ (^2^*J*_C–N2_ = 0.4 Hz and ^3^*J*_C–N3_ = 1.2 Hz), C3′ (^3^*J*_C–N2_ = 1.6 Hz and ^4^*J*_C–N3_ = 0.2 Hz) and C4′ (^4^*J*_C–N2_ = 0.3 Hz) atoms of the adamantane group in 192**a indicated that the initial adamantylation of 37**T occurred on the N2 atom of the tetrazole ring (Table 6 and Scheme 50).

A similar situation with the determination of the adamantylation site in isomer 192**b had arisen. The detected ^13^C–^15^N spin–spin interactions of the ^2^*J*_C1′–N2_ (2.7 Hz), ^3^*J*_C2′–N2_ (1.1 Hz), ^3^*J*_C1′–N3_ (0.3 Hz) and ^4^*J*_C3′–N2_ (0.3 Hz) couplings in the 1D ^13^C NMR spectra of 192**b revealed the attachment of the adamantane fragment to the N1 atom of the tetrazole ring (Table 6 and Scheme 50). However, an analysis of the ^1^H–^15^N coupling constants in the ^15^N-HMBC spectrum (^3^*J*_H2′–N1_ and ^5^*J*_H2′–N3_) and the obtained values of *J*_HN_ from spin-echo experiments in 1D ^1^H NMR did not allow the unambiguous establishment of the structure of 192**b (Table 7 and Scheme 50).

### 4.2. *J*_CN_ and *J*_HN_ coupling constants as an approach for the confirmation of the mechanisms of nitration

**Scheme 51:**
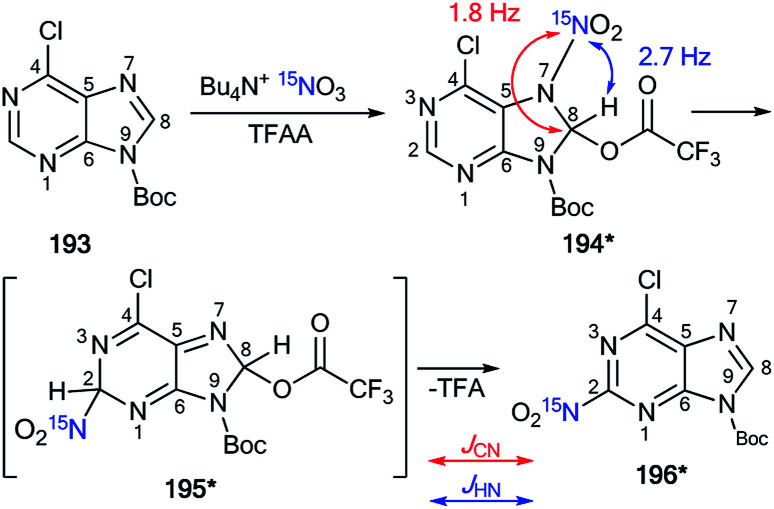
The unusual mechanism of nitration using ^15^N-labeled compounds. The observed *J*_CN_ and *J*_HN_ couplings are shown by red and blue arrows, respectively.

The application of ^15^N-labeled compounds and the measurement of the ^1^H–^15^N and ^13^C–^15^N coupling constants permitted the investigation of the mechanism for nitration in a series of different heterocycles. An unusual method for the incorporation of the nitro group into the core of 6-chloro-9-Boc-purine was found by using a mixture of trifluoroacetic anhydride (TFAA) with labeled tetrabutylammonium nitrate (Bu_4_N^+15^NO_3_^−^) and freezing the nitration reaction (Scheme 51).^63^ The transformation of intermediate 194* was detected at −50 °C by NMR spectroscopy. In the corresponding ^1^H spectrum, the signal of proton H8 was split (^3^*J*_HN_ = 2.7 Hz).

Moreover, in the carbon spectrum of an experiment with Bu_4_N^+15^NO_3_^−^, the ^2^*J*_CN_ (1.8 Hz) coupling was observed for C8, and the signal of the carbon of the carbonyl group for the trifluoroacetoxy fragment was characterized by a ^1^H8–^13^C spin–spin interaction (2.8 Hz). These results unambiguously confirmed the formation of intermediate 194*, which underwent rearrangement into structure 196* at −10 °C. Notably, this process occurred *via* the formation of intermediate 195* (Scheme 51).

**Scheme 52:**
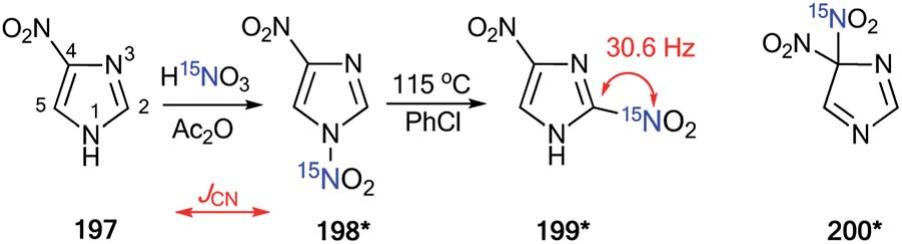
An example of the application of ^15^N-labeled compounds for establishing a nitration mechanism in a series of imidazoles. The observed direct *J*_CN_ coupling is shown by a red arrow.

The use of ^15^N-enriched nitric acid in the nitration of nitroimidazole 197 allowed the detection of the rearrangement occurring in this process.^64^ It was found that the interaction of 197 with ^15^N-nitric acid led to N–^15^NO_2_ dinitroimidazole 198*, which underwent isomerization into compound 199* in chlorobenzene at 115 °C (Scheme 52). It should be noted that the carbon NMR spectrum of dinitroimidazole 199* was only characterized by one direct ^13^C–^15^N coupling constant at the C2 atom with a magnitude of 30.6 Hz (Scheme 52). Thus, the fact that a doublet was observed for the ^13^C resonance at the 2-position not only confirms the assignment of the C-2 resonance but also excluded the formation of intermediate 200* in the process of the isomerization of 198* into 199*. If this transformation included the production of structure 200*, statistically only 50% of the ^15^N would move to the 2-position. Moreover, this result showed that the isomerization included a [1,5]-sigmatropic rearrangement.

### 4.3. Analysis of ^1^H–^15^N and ^13^C–^15^N constants for the confirmation of the structures of amine and imine derivatives

**Scheme 53:**
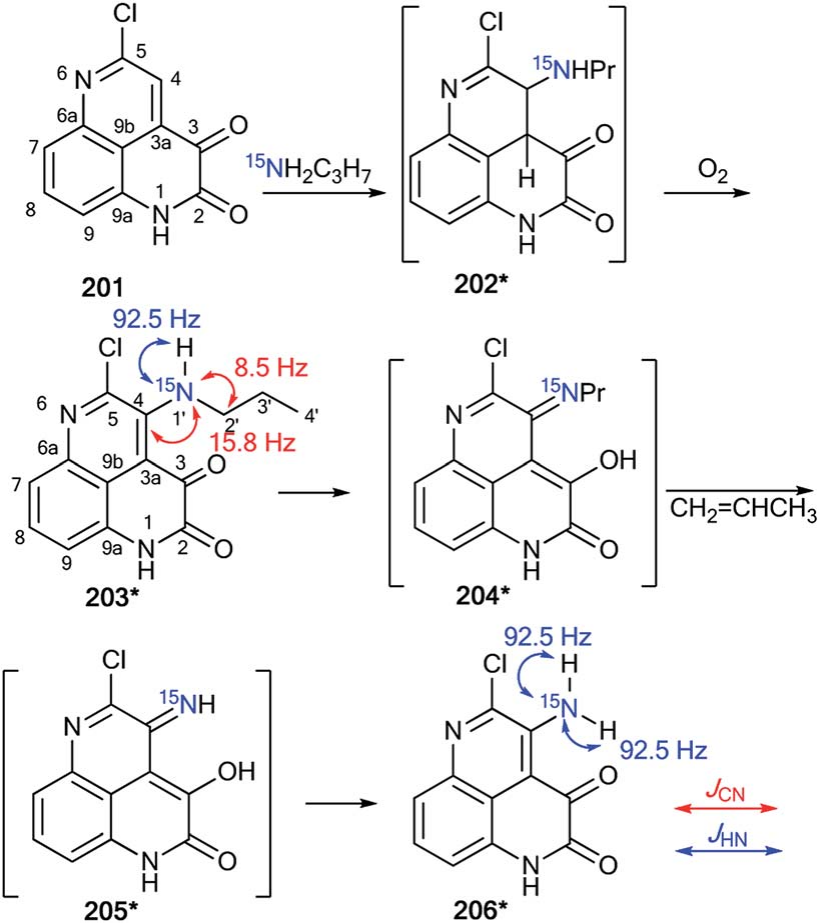
The use of ^15^N-labeled propyl amine to determine the oxidative amination mechanism. The observed direct *J*_CN_ and *J*_HN_ couplings are shown by red and blue arrows, respectively.

The appearance of labeled nitrogen atoms in different amines simplifies the determination of the structures of the reaction products exploiting the analysis of ^13^C–^15^N and ^1^H–^15^N coupling constants. In that vein, the specifics of the interaction of 2,3-diketopyrido[4,3,2-*de*]quinolines 201 with ^15^N-propylamine were studied^65^ (Scheme 53). The ^1^H NMR spectrum of the final product 206* showed signals from two protons that are attached to the ^15^N-labeled atom (^1^*J*_HN_ 92.5 Hz, Scheme 53). These data unambiguously confirmed the production of compounds 206*. Then, the author shed light on the mechanism of this transformation after the isolation of intermediate 203*. The structure of 203* was confirmed by ^1^H and ^13^C NMR spectroscopy. Indeed, the proton spectrum showed that proton H1′ and an *n*-propyl group are attached to the ^15^N atom. The signal of atom H1′ was characterized by a direct ^1^H–^15^N1′ coupling constant with a magnitude of 92.5 Hz (Scheme 53). The aliphatic C2′ and the aromatic C4 are directly linked to the labeled nitrogen atom (^1^*J*_C2′–N1′_ = 8.5 Hz and ^1^*J*_C4–N1′_ = 15.8 Hz, Scheme 53). This result suggested that the transformation of 201 into 206* under the action of ^15^N-propylamine includes an amination–elimination reaction that involves the formation of structures 202*–205* (Scheme 53).

**Scheme 54:**
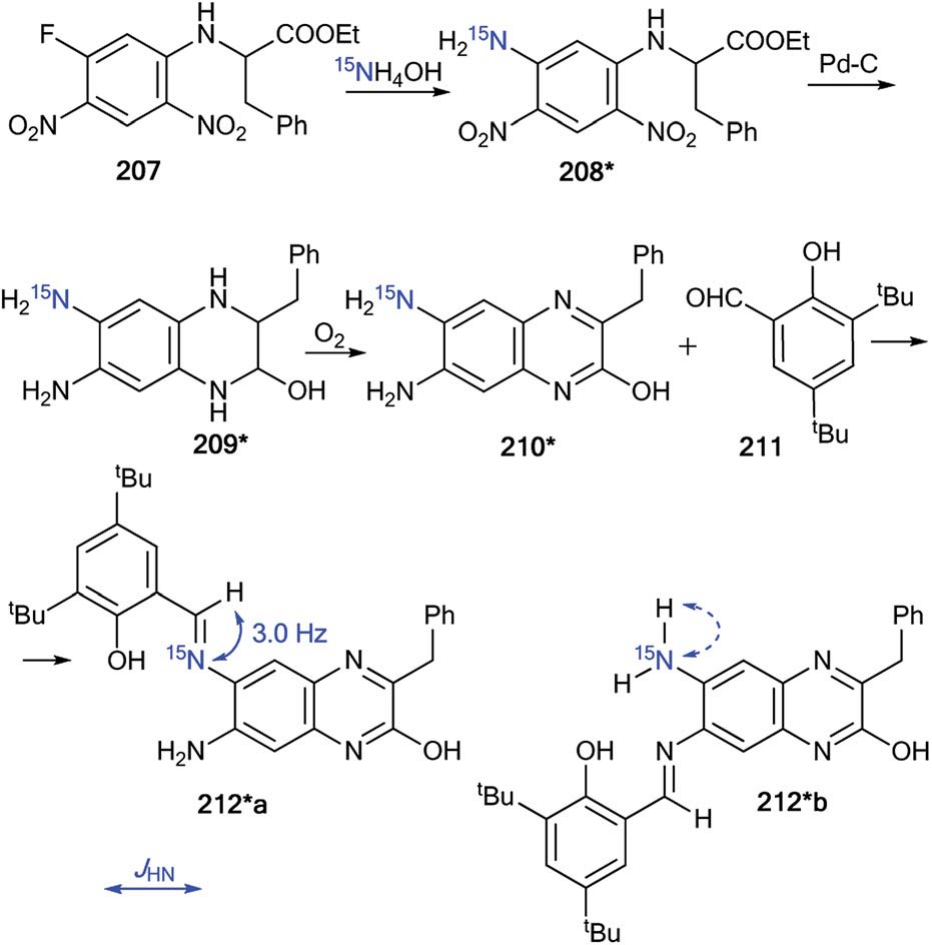
The use of ^15^N-labeled *o*-phenylenediamine for establishing the site of reaction with salicylic aldehyde. The observed ^2^*J*_HN_ coupling is shown by a blue arrow. The expected but unobserved coupling ^1^*J*_HN_ is indicated by a dashed arrow.

The selective incorporation of the ^15^N-label into diamino-2-quinoxalinol 210* permitted the analysis of the ^1^H–^15^N coupling constants for the confirmation of the structure of product 212*, which was isolated from the reaction of 210* with salicylaldehyde 211 (Scheme 54).^66^ A synthesis of compound 210* was based on several steps. Initially, an interaction occurred between compound 207 and ^15^N-labeled ammonium hydroxide (^15^N, 98%), and then 208* was transformed into 209*.

Oxidation of 209* led to diamine 210*, which had two amino groups with different reactivities. Next, reaction of compound 210* with aldehyde 211 could give an imine 212*a or the isomer 212*b. The observation of the ^1^H–^15^N coupling constant for the proton signal of the imine group (^2^*J*_HN_ = 3.0 Hz) confirmed the formation of structure 212*a.

## 5. Conclusion

We have reviewed examples of the incorporation of ^15^N atoms and subsequent analyses of the *J*_HN_ and *J*_CN_ couplings in labeled samples. The above approach can be considered an effective, general, and convenient tool for establishing realistic structures of nitrogen-containing compounds in solution, including mixtures of compounds and equilibrium mixtures. The measurement of the ^1^H–^15^N and ^13^C–^15^N coupling constants allows the investigation of ring-chain tautomerism, the mechanisms of chemical transformation and other structural aspects of azoles, azines, azepines and their fused derivatives. Although this approach is not widely used, ^15^N labeling and estimation of the ^1^H–^15^N and ^13^C–^15^N spin–spin interactions may be a single method that can be efficient for the determination of the molecular structure or method of chemical transformation in the chemistry of poly-nitrogen heterocycles. In structural studies, two types of coupling constants are used, namely, long-range and near (direct, geminal, vicinal) constants. While early publications used only near constants, modern works exploit both long-range and near constants, as well as 2D spectra. For example, 2D ^1^H–^15^N HMBC spectra and spin-echo experiments were used for the determination of the *N*-adamantylation site of azolo-1,2,4-triazines and confirmation of the structure of imidazo[1,2-*a*]pyrimidine. This situation is closely related to the development of the NMR technique. Thus, it can be expected that this approach will be expanded in the chemistry of nitrogen-containing compounds since ^15^N labeling and the analysis of the ^13^C–^15^N and ^1^H–^15^N spin–spin coupling constants shines light on new and known chemical transformations, rearrangements and ring-chain tautomerism in a series of nitrogen heterocycles.

## Conflicts of interest

The authors declare no conflict of interest.

## Supplementary Material
